# Selection of viral capsids and promoters affects the efficacy of rescue of *Tmprss3*-deficient cochlea

**DOI:** 10.1016/j.omtm.2023.08.004

**Published:** 2023-08-11

**Authors:** Ksenia A. Aaron, Katja Pekrun, Patrick J. Atkinson, Sara E. Billings, Julia M. Abitbol, Ina A. Lee, Yasmin Eltawil, Yuan-Siao Chen, Wuxing Dong, Rick F. Nelson, Mark A. Kay, Alan G. Cheng

**Affiliations:** 1 Department of Otolaryngology-Head and Neck Surgery, Stanford University School of Medicine, Stanford, CA 94305, USA; 2 Head and Neck Institute, Cleveland Clinic Foundation, Cleveland, OH 44195, USA; 3 Department of Pediatrics, Stanford University School of Medicine, Stanford, CA 94305, USA; 4 Department of Genetics, Stanford University School of Medicine, Stanford, CA 94305, USA; 5 Department of Otolaryngology-Head and Neck Surgery, Indiana University School of Medicine, Indianapolis, IN 46202, USA

**Keywords:** Tmprss3, cochlea, hair cells, hearing loss, supporting cells, gene therapy

## Abstract

Adeno-associated virus (AAV)-mediated gene transfer has shown promise in rescuing mouse models of genetic hearing loss, but how viral capsid and promoter selection affects efficacy is poorly characterized. Here, we tested combinations of AAVs and promoters to deliver *Tmprss3*, mutations in which are associated with hearing loss in humans. *Tmprss3*^*tm1/tm1*^ mice display severe cochlear hair cell degeneration, loss of auditory brainstem responses, and delayed loss of spiral ganglion neurons. Under the ubiquitous CAG promoter and AAV-KP1 capsid, *Tmprss3* overexpression caused striking cytotoxicity *in vitro* and *in vivo* and failed to rescue degeneration or dysfunction of the *Tmprss3*^*tm1/tm1*^ cochlea. Reducing the dosage or using *AAV-DJ-CAG-Tmprss3* diminished cytotoxicity without rescue of the *Tmprss3*^*tm1/tm1*^ cochlea. Finally, the combination of AAV-KP1 capsid and the EF1α promoter prevented cytotoxicity and reduced hair cell degeneration, loss of spiral ganglion neurons, and improved hearing thresholds in *Tmprss3*^*tm1/tm1*^ mice. Together, our study illustrates toxicity of exogenous genes and factors governing rescue efficiency, and suggests that cochlear gene therapy likely requires precisely targeted transgene expression.

## Graphical abstract

**Figure:**
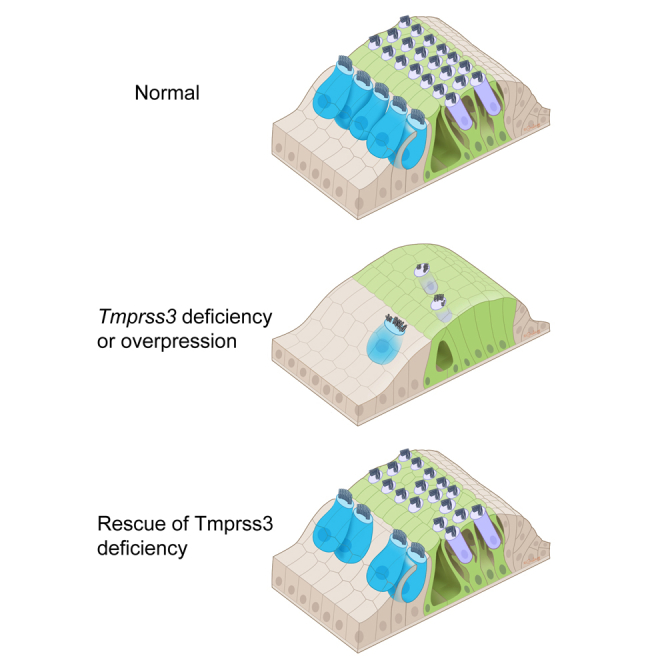

## Teaser

Cheng and colleagues show that selection of viral capsids and promoters can affect the toxicity of exogenous gene transfer of mouse Tmprss3 and its efficacy of rescue of hair cell loss and hearing loss caused by Tmprss3 deficiency. Tmprss3 deficiency causes hair cell loss and hearing loss in humans.

## Introduction

Nearly 1 in 500 children in the United States is born with hearing loss, 65% of which are caused by genetic mutations.^1^ More than 70% of genetic hearing loss is attributed to autosomal recessive, nonsyndromic deafness (ARNSD) mutations,^2^ up to 10% of which are caused by transmembrane protease serine 3 (*TMPRSS3*) mutations. Missense mutations of *TMPRSS3* cause variable onset and degrees of sensorineural hearing loss, leading to prelingual (DFNB10) or postlingual (DFNB8) ARNSD.^2^^,^^3^^,^^4^ As one of the most common causal genes of hearing loss among adult cochlear implant recipients,^5^ *TMPRSS3* mutations currently lack a biological treatment that prevents or reverses the course of disease.

**Figure 1:**
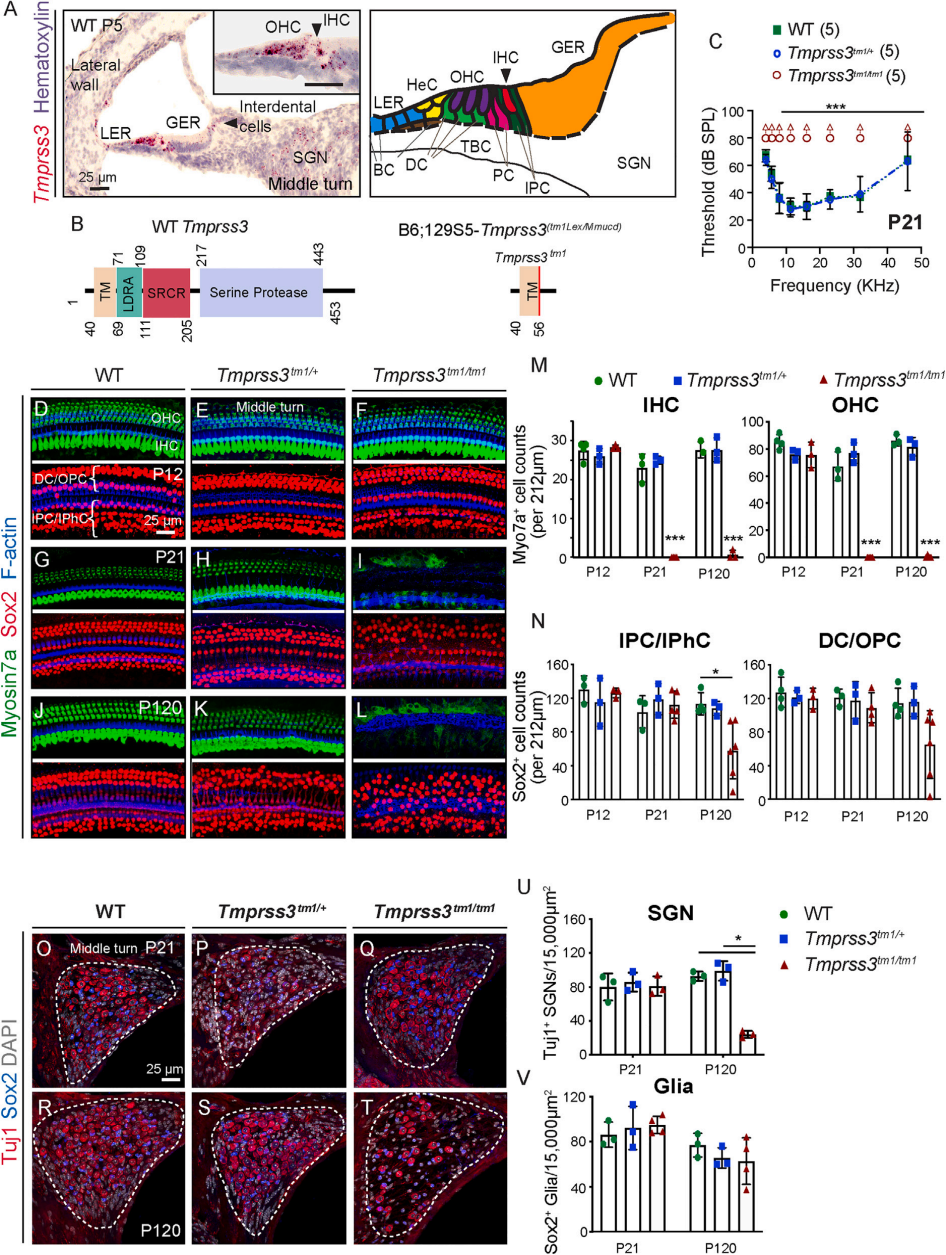
Tmprss3 deficiency causes cochlear hair cell degeneration and hearing loss (A) *In situ* hybridization (RNAScope) of P5 wild-type (WT) cochlea (middle turn and counterstained with hematoxylin) revealed *Tmprss3* mRNA expression in hair cells and supporting cells, interdental cells, inner phalangeal cells, lesser epithelial ridge, outer sulcus, and Rosenthal’s canal. Schematic depicting hair cell and supporting cell subtypes. (B) The TMPRSS3 protein consists of 453 amino acids, with a transmembrane (TM) domain, a low-density lipoprotein receptor class A (LDRA), a scavenger receptor cysteine-rich domain (SRCR), and a C-terminal serine protease. The mutation was generated by targeted mutation through homologous recombination in exon 1. (C) At P21, *Tmprss3*^*tm1/+*^ littermates had ABR thresholds that were indistinguishable from WT littermates, whereas *Tmprss3*^*tm1/tm1*^ mice demonstrated no ABR responses across all frequencies tested. (D–F) Immunostaining of P12 cochleae showed no loss or disorganization of hair cells and supporting cells among WT, *Tmprss3*^*tm1/+*^, and *Tmprss3*^*tm1/tm1*^ littermates prior to the onset of hearing. (G–L) Substantial inner and outer hair cell loss and disorganized supporting cells were observed in the P21 and P120 *Tmprss3*^*tm1/tm1*^ mice. No cell loss in WT or *Tmprss3*^*tm1/+*^ cochleae. (M–N) Quantification in the middle cochlear turn showing significant loss of hair cells in P21 and P120 *Tmprss3*^*tm1/tm1*^ cochleae and medial supporting cell loss at P120. (O–Q) Cross sections of Rosenthal’s canal at P21 showing no spiral ganglia neuron degeneration. (R–T) At P120, there was a noticeable loss of spiral ganglion neurons in the *Tmprss3*^*tm1/tm1*^ cochleae. (U–V) Quantitative analysis showing a significant loss of TuJ1^+^ spiral ganglion neurons, but not Sox2^+^ glial cells, in P120 *Tmprss3*^*tm1/tm1*^ cochleae. Data shown as mean ± SD. ∗p < 0.05, ∗∗p < 0.01, ∗∗∗p < 0.001. Two-way ANOVA with Tukey’s multiple comparison. n = 3–6. IHC, inner hair cell; OHC, outer hair cell; DC, Deiters’ cell; IPhC, inner phalangeal cell; IPC, inner pillar cell; OPC, outer pillar cell; SGN, spiral ganglion neuron.

The mammalian cochlea is composed of distinct sensory and non-sensory cell types that are all essential for auditory function (Figure 1A). As mechanoreceptors, hair cells convert mechanical stimuli to electrical signals, are intercalated by supporting cells, and relay auditory input centrally via spiral ganglion neurons. Mice deficient in *Tmprss3* (Y260X), where a nonsense mutation results in a truncated protease domain, exhibit normal cochlear development followed by rapid hair cell degeneration during the onset of hearing at postnatal day (P) 12.^6^ This leads to a complete loss of auditory brainstem responses (ABRs), implicating a requirement of *Tmprss3* for hair cell survival and cochlear function.

Previous histological and single-cell RNA sequencing studies showed that *Tmprss3* is broadly expressed in sensory hair cells (outer and inner), supporting cells (Deiters’, pillar, and inner phalangeal cells) and a subset of spiral ganglion neurons in the embryonic and postnatal cochleae.^7^^,^^8^^,^^9^^,^^10^^,^^11^^,^^12^ *Tmprss3* encodes a serine protease and has been postulated to regulate ionic homeostasis of the cochlea.^6^^,^^7^^,^^8^^,^^9^^,^^13^ Both epithelial sodium channels (ENaCs) and calcium-activated potassium (BK) channels are candidate downstream targets of *Tmprss3*,^9^^,^^10^^,^^11^^,^^12^^,^^13^ yet loss of function of ENaC does not cause hearing loss in humans, while deletion of BK channels leads to hearing loss only after 8 weeks of age in mice, well after the onset of hearing.^14^^,^^15^ At present, the exact function of *Tmprss3* and the pathogenesis of DFNB8/10 are unclear.

In the inner ear, adeno-associated virus (AAV) capsids have demonstrated low immunogenicity and variable degrees of transduction efficacy in multiple cell types.^16^ To rescue mouse models of hearing loss caused by hair cell mutations, independent studies have used AAV capsid types exhibiting high tropism for cochlear hair cells and employed ubiquitous promoters to overexpress genes of interest.^17^^,^^18^^,^^19^^,^^20^^,^^21^^,^^22^^,^^23^^,^^24^ However, whether this approach is effective for mutations affecting multiple cochlear cell types and whether selection of viral capsids and promoters affects the overall efficacy of rescue are not known.

Here, we used recombinant AAV (rAAV) vectors in an attempt to restore *Tmprss3* expression and function in *Tmprss3*^*tm1*^ knockout mice (*Tmprss3*^*tm1/tm1*^). Our studies demonstrate that *Tmprss3* is required for hair cell survival and auditory function. During rescue experiments, we discovered that the engineered KP1 capsid was capable of transducing cochlear cells with high efficiency.^25^ However, when administering the *Tmprss3* transgene under control of the ubiquitous promoter (CAG)^26^ we observed cytotoxicity both *in vitro* and *in vivo*. Treatment with a selective viral capsid (AAV-DJ^27^) and decreasing vector dose reduced toxicity but failed to prevent hair cell loss or auditory dysfunction. In contrast, by using the EF1α core promoter,^28^ hair cell survival and auditory function were partially rescued in *Tmprss3*^*tm1*^ knockout mice. Together, we have established that the input capsid, promoter, and vector dose dictate cytotoxicity and efficacy in rescuing the *Tmprss3*-deficient cochlea.

## Results

### *Tmprss3* is expressed in multiple cochlear cell types

To characterize *Tmprss3* expression in the mouse cochlea, we performed RNAScope *in situ* hybridization. In the embryonic (E) day 18, postnatal 1- and 5-day-old (P1 and 5) wild-type cochlea, *Tmprss3* mRNA was robustly expressed in the organ of Corti, including the inner hair cells, outer hair cells, and supporting cell subtypes (Figures 1A, S1A, and S1B). We did not observe a tonotopic gradient in expression. To a lesser extent, *Tmprss3* transcripts were also detected in the greater epithelial ridge, lesser epithelial ridge, interdental cells, lateral cochlear wall, and select spiral ganglion neurons (Figure 1A). This is consistent with previous single-cell RNA sequencing and *in situ* hybridization data.^11^^,^^12^^,^^25^^,^^29^ We next assessed the effects of *Tmprss3* deficiency by examining the *Tmprss3*^*tm1*^ mouse line (Figure 1B), which is a knockout model generated through targeted mutation by homologous recombination in exon 1.^30^ Using probes designed to detect the deleted sequences (BaseScope), we found that *Tmprss3-exon1-2* transcripts were absent in the P1 *Tmprss3*^*tm1/tm1*^ cochlea, whereas *Tmprss3* transcripts remained detectable in different cell types in the P1 wild-type cochlea (Figures S1E and S1F). These results indicate that *Tmprss3* mRNA expression is effectively abolished in the *Tmprss3*^*tm1/tm1*^ cochlea.

### *Tmprss3* deficiency leads to hair cell degeneration and cochlear dysfunction

To determine whether *Tmprss3* is required for cochlear maturation, we first examined cochleae in P5 *Tmprss3*^*tm1/tm1*^ mice and found no evidence of hair cell or supporting cell loss with cell counts comparable to those of *Tmprss3*^*tm1/+*^ and wild-type mice (Table S1). Similarly, each turn of the P12 and P13.5 *Tmprss3*^*tm1/tm1*^ cochleae showed comparable counts and organization of sensory hair cells and supporting cells to *Tmprss3*^*tm1/+*^ and wild-type cochleae (Figures 1D–1F, 1M, S1K, and S1L; Table S1), suggesting that *Tmprss3* is not required for hair cell patterning or survival from P5 to 13.5.

Shortly after the onset of hearing around P14–14.5, *Tmprss3*^*tm1/tm1*^ cochleae showed rapid and extensive degeneration of both inner and outer hair cells (Figures S1M–S1P), corroborating previous results in *Tmprss3* (Y260X) mice.^6^ By P21, all Myosin7a^+^ hair cells had significantly degenerated (p < 0.0001) in the *Tmprss3*^*tm1/tm1*^ cochleae, whereas wild-type and *Tmprss3*^*tm1/+*^ cochleae showed similar cell counts and organization (Figures 1G–1I and 1M; Table S1). As expected from the severe hair cell loss, P21 *Tmprss3*^*tm1/tm1*^ mice exhibited no detectable ABR at any frequency tested (Figure 1C), whereas both wild-type and *Tmprss3*^*tm1/+*^ mice displayed robust responses. These results demonstrate that *Tmprss3* is required for hair cell survival and cochlear function after the second postnatal week.

### Degeneration of supporting cells and spiral ganglion neurons in mature *Tmprss3*^*tm1/tm1*^ cochleae

In the juvenile and mature *Tmprss3*^*tm1/tm1*^ cochlea, sensory hair cell loss was the prominent feature. Sox2^+^ supporting cell subtypes, which expressed *Tmprss3* mRNA between E18.5 and P5, appeared disorganized in the P21 *Tmprss3*^*tm1/tm1*^ cochlea, likely as a result of severe hair cell loss (Figures 1G–1I). However, we did not detect significant degeneration of supporting cells (Figure 1N; Table S1) or spiral ganglion neurons at this age (Figures 1O–1Q and 1U; Table S1). By P120, in addition to hair cell loss, there was a moderate and variable degree of supporting cell loss in the *Tmprss3*^*tm1/tm1*^ cochleae (Figures 1J–1L and 1N). Relative to P21 *Tmprss3*^*tm1/tm1*^ cochleae, there were significantly fewer supporting cells in the apical and middle turns of the P120 *Tmprss3*^*tm1/tm1*^ cochlea (Table S1).

In the Rosenthal canal, no degeneration of spiral ganglion neurons or glia was observed in P21 *Tmprss3*^*tm1/tm1*^ mice (Figures 1O–1Q, 1U, and 1V; Table S1). However, there were significantly fewer Tuj1^+^ spiral ganglion neurons, but not Sox2^+^ glia, in each turn of the P120 *Tmprss3*^*tm1/tm1*^ cochlea relative to age-matched *Tmprss3*^*tm1/+*^ and wild-type controls (Figures 1R–1V; Table S1). Together, these results indicate that *Tmprss3* deficiency causes delayed loss of cochlear supporting cells and spiral ganglion neurons in the adult cochlea.

### AAV-KP1 transduces multiple cochlear cell types with high efficacy

**Figure 2:**
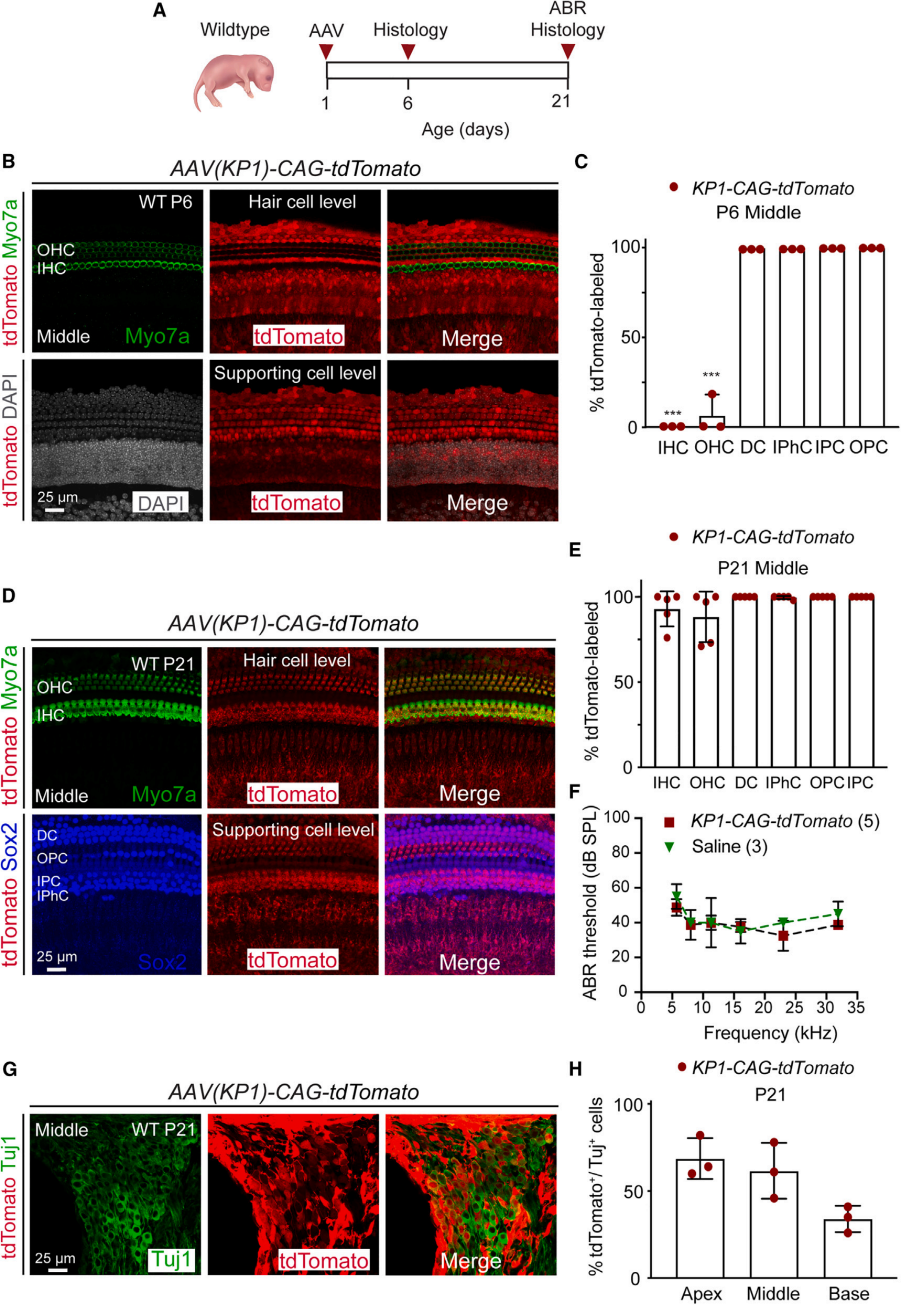
Tropism and efficacy of *AAV-KP1-CAG-tdTomato* in the cochlea (A) Schematic showing AAV injection at P1 in WT pups and examination at P6 and P21. (B) Robust tdTomato expression in supporting cells (bottom) but not hair cells (top) in the P6 cochlea (middle turn shown). (C) Quantification of labeled hair cells and supporting cells. (D) Robust tdTomato expression in both hair cells and supporting cells (top and bottom) at P21. (E) Quantitative analysis of tdTomato-labeled hair cells and supporting cell subtypes. (F) Both saline- and *AAV-KP1-CAG-tdTomato*-injected animals showed normal ABR thresholds at P21. (G) Only a subset of Tuj1^+^ spiral ganglion neurons were tdTomato labeled at P21. (H) Spiral ganglion neurons were partially transduced in all three cochlear turns, with the apex showing the highest rate. Data shown as mean ± SD. ∗∗∗p < 0.001. Two-way ANOVA with Tukey’s multiple comparison. n = 3–5.

As *Tmprss3* is expressed in multiple cochlear cell types, we postulated that a gene delivery approach that is ubiquitous and efficient would be needed to rescue the phenotype caused by its deficiency. The AAV-KP1 capsid was obtained from a screen of a shuffled AAV capsid library on primary human islet cells and shown to have transduction efficacy across multiple murine and human cell lines comparable with or higher than the AAV-DJ capsid.^31^^,^^32^ However, neither capsid had been systematically evaluated for its ability to transduce inner ear cell types. To characterize the tropism and efficacy of both chimeric capsids in the cochlea, we generated rAAV vectors carrying the tdTomato reporter expressed under a CAG promoter and packaged them using KP1 and DJ capsids (*AAV-KP1-CAG-tdTomato* and *AAV-DJ-CAG-tdTomato*). Viral capsids were injected via a posterior semicircular canal approach into P1 pups (1 μL injected over 3 min, 1.0 × 10^9^ vector genomes [vg]) (Figure 2A).

Five days after injection with *AAV-KP1-CAG-tdTomato* (P6), no or minimal tdTomato expression was detected in inner and outer hair cells (apex, 0.0% and 0.0%; mid, 0.0% and 6.6% ± 11.5%; base, 0.0% and 0.0%, respectively), whereas supporting cell subtypes showed robust expression (Figures 2B and 2C; Table S2). Twenty days post injection (P21), there was broad tdTomato expression in both inner and outer hair cells (>90.4% ± 8.3% and 84.0% ± 14.0%, respectively) across all three turns, as well as sustained labeling of supporting cells (>99.6% in all three turns) (Figures 2D and 2E; Table S2). Transduction efficacy of spiral ganglion neurons at P21 was found to be lower than those of both hair cells and supporting cells (68.7% ± 11.7% apical, 61.7% ± 16.0% middle, and 34.0% ± 7.5% basal turns; Figures 2G and 2H; Table S2). The P21 contralateral, control cochleae showed no tdTomato expression in inner and outer hair cells with only occasional transduction of supporting cells in the basal turn in three of four animals (Deiters’ cells, 26.6% ± 31.1%; outer pillar cells, 25.5% ± 24.3%; inner pillar cells, 15.8% ± 14.6%; and inner phalangeal cells, 36.2% ± 28.4%; Figures S2A–S2D; Table S2).

After injection with *AAV-DJ-CAG-tdTomato* capsid at P1, P21 wild-type mice demonstrated tdTomato-labeled hair cells and supporting cells, albeit at lower rates than with the KP1 capsid (Figures S2E and S2F). In the middle turn, the transduction rates were 65.6% ± 29.3% in inner hair cells, 30.0% ± 19.5% outer hair cells, 54.7% ± 5.0% Deiters’ cells, 48.7% ± 15.9% inner pillar cells, 52.3% ± 30.2% outer pillar cells, and 76.4% ± 25.0% inner phalangeal cells (Figure S2F; Table S2). Similar to saline-injected animals, those injected with rAAV packaged with either KP1 or DJ capsids exhibited no detectable ABR threshold shifts at P21 (Figure 2F and S2G). Together, these data indicate that both AAV-KP1 and AAV-DJ capsids transduce cochlear hair cells and supporting cells with no adverse effects on cell survival or cochlear function, with the former more efficiently transducing cochlear cells *in vivo*. Furthermore, these data show the temporal differences in tdTomato expression where there is a delay in the onset of *KP1-CAG-tdTomato* expression in hair cells compared to that of *DJ-CAG-tdTomato* expression.

### Overexpression of *Tmprss3* is cytotoxic *in vitro* and *in vivo*

**Figure 3:**
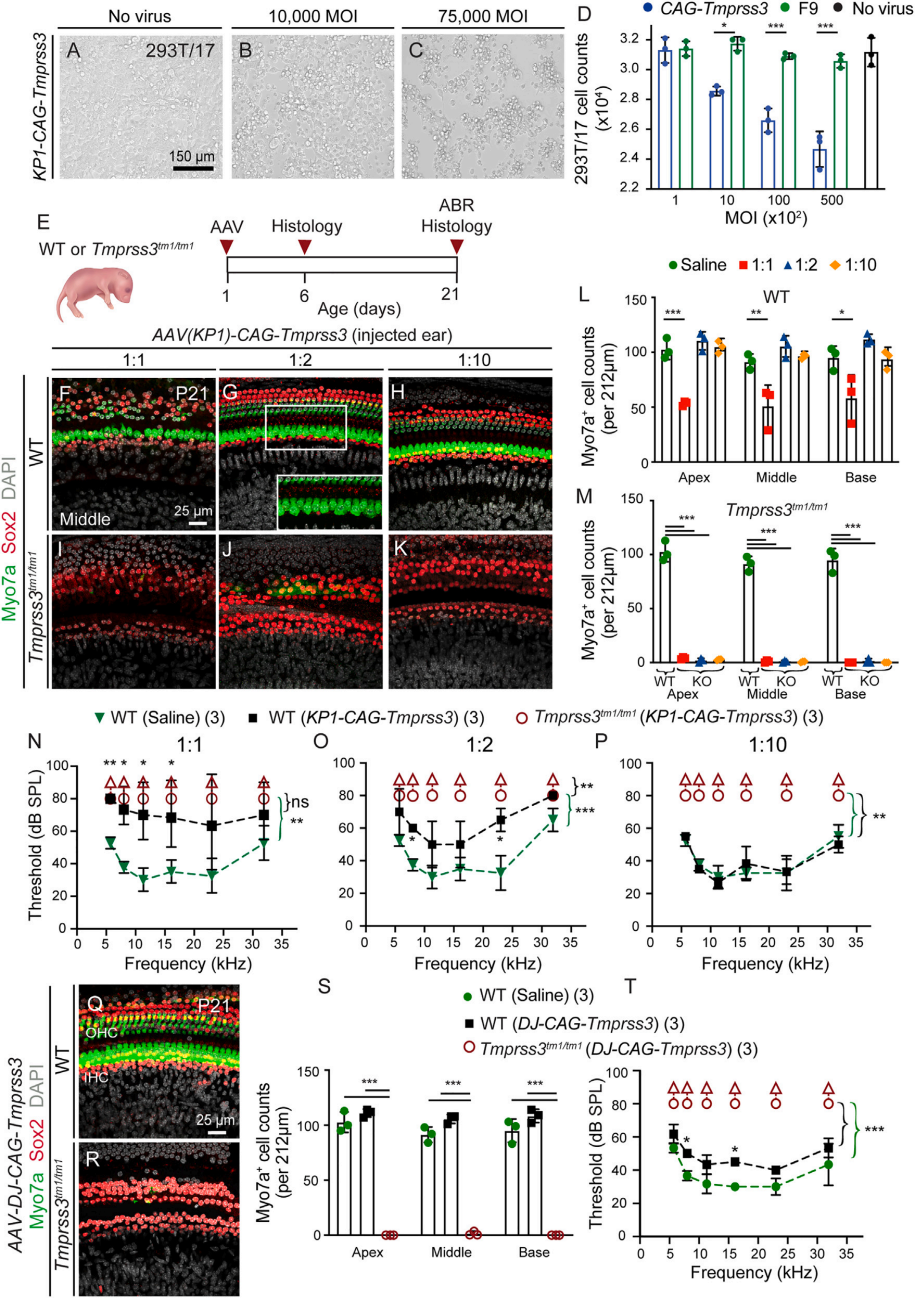
Exogenous TMPRSS3 is cytotoxic *in vitro* and *in vivo* (A–C) Transduction of 293T/17 (human embryonic kidney) cells with *AAV-KP1-CAG-Tmprss3* resulted in cell death in a dose-dependent manner, whereas none was observed in no-virus controls. (D) Proliferation assay demonstrating that increasing the MOI with *AAV-KP1-CAG-Tmprss3* significantly decreased 293T/17 cell counts. Controls using huF9-expressing rAAV and no virus showed higher viability. (E) Schematic showing the timeline of injection of viral vectors into WT or mutant (*Tmprss3*^*tm1/tm1*^) cochleae and subsequent examination at P6 and P21. (F) At high titers (1:1, 2.0 × 10^8^ vg), *AAV-KP1-CAG-Tmprss3* caused degeneration of OHCs and swelling of IHCs. (G) A lower titer (1:2, 1.0 × 10^8^ vg) did not cause hair cell degeneration, although IHCs still appeared swollen (inset). (H) No degeneration was observed at a 1:10 dilution (2.0 × 10^7^ vg). (I–K) Hair cell degeneration was not prevented in P21 *Tmprss3*^*tm1/tm1*^ cochleae with any titers of *AAV-KP1-CAG-Tmprss3* tested. (L) Quantification at P21 showing significant hair cell loss in all three turns of the cochlea at 1:1, but not other dilutions, of *AAV-KP1-CAG-Tmprss3*. (M) Hair cells degenerated in each turn of P21 *Tmprss3*^*tm1/tm1*^ cochleae injected with any titers of *AAV-KP1-CAG-Tmprss3*, resulting in significantly fewer hair cells than saline-injected, WT controls. (N) ABR thresholds were significantly higher in the ears of P21 WT animals injected with the full 1:1 titer *AAV-KP1-CAG-Tmprss3* relative to saline-injected WT controls. (O and P) Some elevation of ABR thresholds was observed at a 1:2 dilution, and no changes were observed at a 1:10 dilution. *Tmprss3*^*tm1/tm1*^ mice displayed no ABR responses across all three titers. (Q) No cell loss was detected in the P21 WT cochlea after *AAV-DJ-CAG-Tmprss3* had been injected at P1 (2.0 × 10^8^ vg), although IHC appeared swollen. (R) Sensory hair cell loss at P21 was not prevented by *AAV-DJ-CAG-Tmprss3* in *Tmprss3*^*tm1/tm1*^ mice. (S) Hair cell counts in saline- and *AAV-DJ-CAG-Tmprss3*-injected WT cochleae and *AAV-DJ-CAG-Tmprss3*-injected *Tmprss3*^*tm1/tm1*^ cochleae. (T) WT ears injected with *AAV-DJ-CAG-Tmprss3* showed elevated ABR thresholds at 8 and 16 kHz. *Tmprss3*^*tm1/tm1*^ ears treated with *AAV-DJ-CAG-Tmprss3* had no ABR responses. Data shown as mean ± SD. ∗p < 0.05, ∗∗p < 0.01, ∗∗∗p < 0.001. Two-way ANOVA with Tukey’s multiple comparison. n = 3.

To begin examining the effects of exogenous *Tmprss3*, we generated a KP1 capsid-packaged rAAV vector expressing the mouse *Tmprss3* construct under the control of a CAG promoter. During rAAV production, detachment and death of producer cells (HEK293T/17) were noted, requiring a shortening of the incubation period prior to rAAV harvest (30 h instead of 60–72 h). Cell proliferation decreased with increasing multiplicity of infection (MOI) of *AAV-KP1-CAG-Tmprss3* but not for a control rAAV-factor IX expression vector or for non-transduced cells (Figures 3A–3D). Similarly, dose-dependent cytotoxicity was found with *rAAV-KP1-CAG-Tmprss3* using HeLa cells (Figures S3A–S3D).

To determine whether AAV-related toxicity extends to cochlear tissues, we next administered *AAV-KP1-CAG-Tmprss3* (2.0 × 10^8^ vg) into the cochlea in P1 *Tmprss3*^*tm1/tm1*^ and wild-type mice (Figure 3E). In the wild-type cochlea, no degeneration or disorganization of hair cells was observed at P6 (Figures S3E and S3F), while striking disorganization and degeneration of hair cells was noted at P21 (Figure 3F). There were significantly fewer Myo7a^+^ hair cells in each cochlear turn (apex, 53.3 ± 2.1; middle, 51.0 ± 19.1; and base, 58.3 ± 21.1) than in saline-injected controls (apex, 102.7 ± 9.2; middle, 91.3 ± 7.0; and base, 95.0 ± 10.5) (p < 0.05; Figure 3L; Table S3). Sox2^+^ supporting cells also appeared disarrayed, but no degeneration was detected at P21 (Figure 3F; Table S3). As controls, no hair cell or supporting cell degeneration was detected in the contralateral ears at P21 (Figures S3G–S3I and S3M). Reducing the injected viral titers 1:2 and 1:10 (1.0 × 10^8^ and 2.0 × 10^7^ vg, respectively) prevented hair cell degeneration in the P21 wild-type cochleae, although inner hair cells appeared swollen after delivery of the former (1:2) titer rAAV (Figures 3G–3H and 3L). *AAV-KP1-CAG-Tmprss3* at full or reduced titers failed to prevent degeneration of hair cells in P21 *Tmprss3*^*tm1/tm1*^ mice (Figures 3I–3K, 3M, and S3J–S3M).

Additionally, *AAV-KP1-CAG-Tmprss3* injection resulted in elevated ABR thresholds that were significantly higher than those of saline-injected P21 wild-type animals (p < 0.01; Figure 3N) and non-injected ears (Figure S3N). Halving the titers lessened, and a 10-fold dilution prevented, ABR threshold shifts (Figures 3O–3P). However, *AAV-KP1-CAG-Tmprss3* at full or reduced titers failed to rescue ABR thresholds in *Tmprss3*^*tm1/tm1*^ mice (Figures 3N–3P). Together, these results indicate that exogenous *Tmprss3* causes cytotoxicity *in vitro* and *in vivo* and that decreasing transduction reduced toxicity but failed to prevent *Tmprss3* deficiency-induced hair cell loss and auditory dysfunction.

### Exogenous *Tmprss3* toxicity is associated with multiple AAV capsid types

To verify that cytotoxicity can be reduced by decreasing transduction rates, we also used AAV-DJ to overexpress *Tmprss3 in vivo*. *AAV-DJ-CAG*-*Tmprss3* (2.0 × 10^8^ vg) was administered to P1 *Tmprss3*^*tm1/tm1*^ and wild-type mice. In the injected P21 wild-type cochlea, no hair cell loss was detected (Figures 3Q, 3S, S4, and S4B), while a small but significant ABR threshold shift across several frequencies was observed, suggesting some cytotoxicity similar to the lower titers of *AAV-KP1-CAG-Tmprss3* (p < 0.05; Figure 3T). Moreover, *AAV-DJ-CAG*-*Tmprss3* administration failed to prevent hair cell loss or ABR threshold shifts in *Tmprss3*^*tm1/tm1*^ mice (Figures 3R–3T, S4C, and S4D). Thus, *Tmprss3*-related cytotoxicity is likely dependent on transduction efficiency and can occur with both AAV-KP1 and AAV-DJ viral capsids. The presence of normal numbers of hair cells in *DJ-CAG-Tmprss3*-transduced wild-type cochleae as well as the presence of ABR thresholds, albeit significantly elevated, in these mice suggest that functional hair cells are present. Thus, viral transduction may be affecting supporting cells or cells outside of the organ of Corti. Additionally, using a capsid with lower transduction efficacy failed to prevent hair cell degeneration and auditory dysfunction caused by *Tmprss3* deficiency.

### *AAV-KP1-EF1α-Tmprss3* gene vector is not cytotoxic *in vitro* or *in vivo*

**Figure 4:**
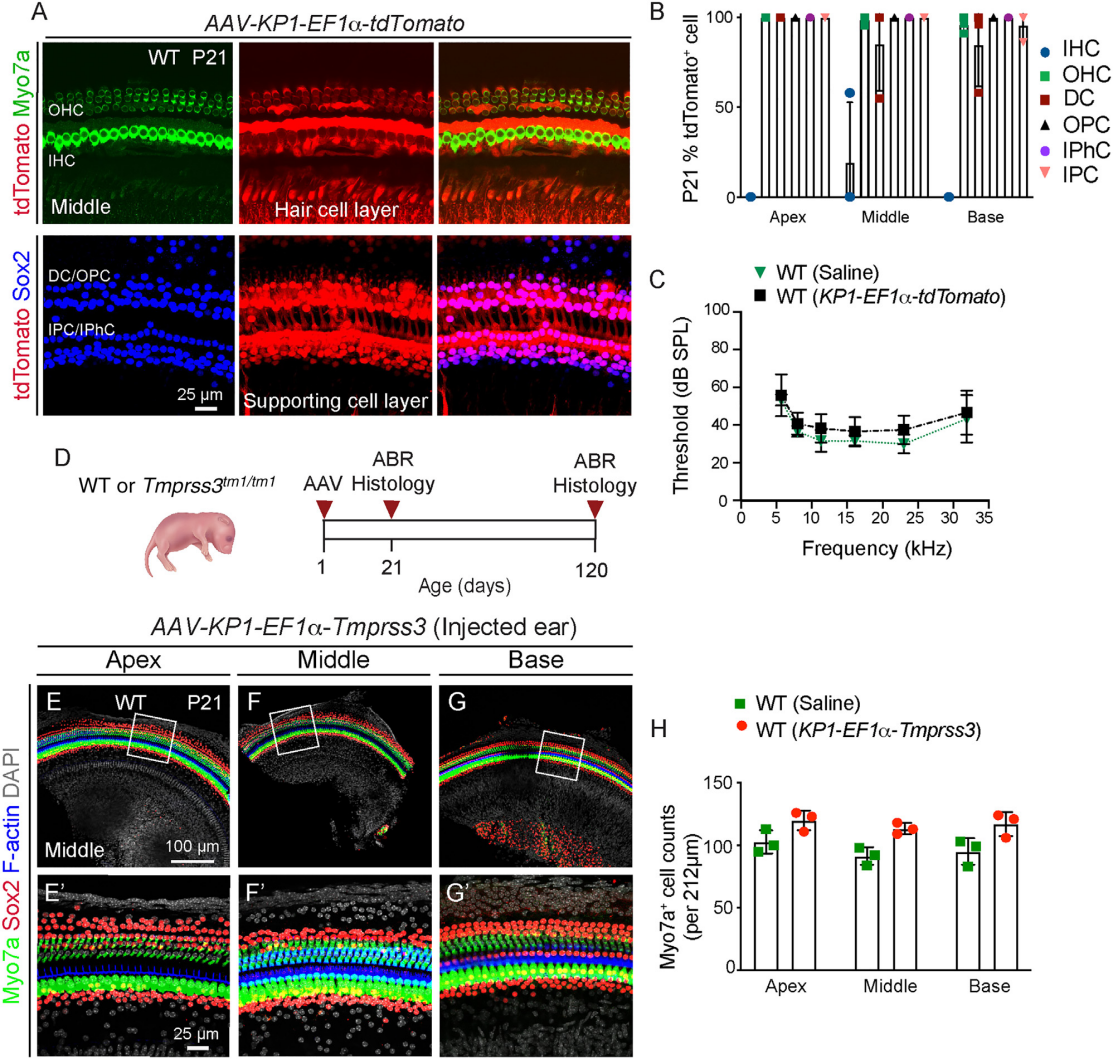
*AAV-KP1-EF1α-Tmprss3* is not cytotoxic in the cochlea *in vivo* (A) After injection of *AAV-KP1-EF1α-tdTomato* (1.0 × 10^9^ vg/mL) at P1, there was robust tdTomato expression in OHCs (top) and supporting cell subtypes (lower) at P21 (middle turn shown). (B) Most OHCs and supporting cell subtypes and almost no IHCs were tdTomato labeled. (C) Relative to saline-injected ears, there were no detectable shifts in ABR thresholds in the *AAV-KP1-EF1α-tdTomato*-injected ears at P21. (D) Schematic showing the timeline of injection of *AAV-KP1-EF1α-Tmprss3* into WT or mutant (*Tmprss3*^*tm1/tm1*^) cochleae and subsequent examination at P21 and P120. (E–G) *AAV-KP1-EF1α-Tmprss3* (6.5 × 10^8^ vg) injected into P1 WT mice did not cause any loss of sensory cells or supporting cells across all three turns at P21. (E′–G′) High-magnification images from (E–G). (H) Quantification of hair cells in saline- and *AAV-KP1-EF1α-Tmprss3*-injected WT cochleae. Data shown as mean ± SD. Two-way ANOVA with Tukey’s multiple comparison. n = 3.

The promoter EF1α has been demonstrated to drive lower transgene expression relative to the CAG promoter in various tissue types.^33^^,^^34^^,^^35^ To determine whether this promoter can help abolish cytotoxicity, we generated an AAV-KP1 vector packaging *Tmprss3* cDNA under the control of an EF1α core promoter. We next tested the transduction efficacy of *AAV-KP1-EF1α-tdTomato in vivo*. Five days post injection into wild-type cochleae, most inner and outer hair cells (apex, 91.5% ± 12.3% and 97.9% ± 3.4%; middle, 60.9% ± 36.8% and 75.4% ± 39.5%, base, 70.0% ± 19.6% and 79.3% ± 23.4%) and almost all supporting cell subtypes expressed tdTomato (Figures S4A and S4B). At 20 days post injection, both outer hair cells and supporting cells, but not inner hair cells, remained highly transduced (>95.8% in all three turns; Figures 4A and 4B; Table S3). Similar to *AAV-KP1-CAG-tdTomato*, no ABR threshold shifts were detected after *AAV-KP1-EF1α-tdTomato* administration (Figure 4C). In contralateral control cochleae, tdTomato expression was observed in some supporting cells of the basal turn (Deiters’ cells, 10.3% ± 17.9%; outer pillar cells, 10.7% ± 17.6%; inner pillar cells, 37.8% ± 41.6%; and inner phalangeal cells, 18.1% ± 16.8%; Table S2) but not in middle or apical turns.

Unlike *AAV-KP1-CAG-Tmprss3*, the *AAV-KP1-EF1α-Tmprss3* construct did not diminish proliferation of 293T/17 cells at various MOIs, with rates similar to those of no-virus controls and those transduced with a control rAAV-factor IX prep (Figures S5C–S5F). Collectively, these results suggest that the use of the EF1α promoter abolished the cytotoxicity of exogenous *Tmprss3 in vitro.*

We next administered the *AAV-KP1-EF1α-Tmprss3* (6.5 × 10^8^ vg) vector into P1 wild-type mice (Figure 4D). At P21, we did not detect any degeneration of hair cells or supporting cells in any cochlear turns (Figures 4E–4H; Table S3). Collectively, these results suggest that the use of the EF1α promoter abolished the cytotoxicity of exogenous *Tmprss3 in vitro* and *in vivo*.

### *AAV-KP1-EF1α-Tmprss3* partially prevents cochlear degeneration and auditory dysfunction in *Tmprss3*^*tm1/tm1*^ mice

**Figure 5:**
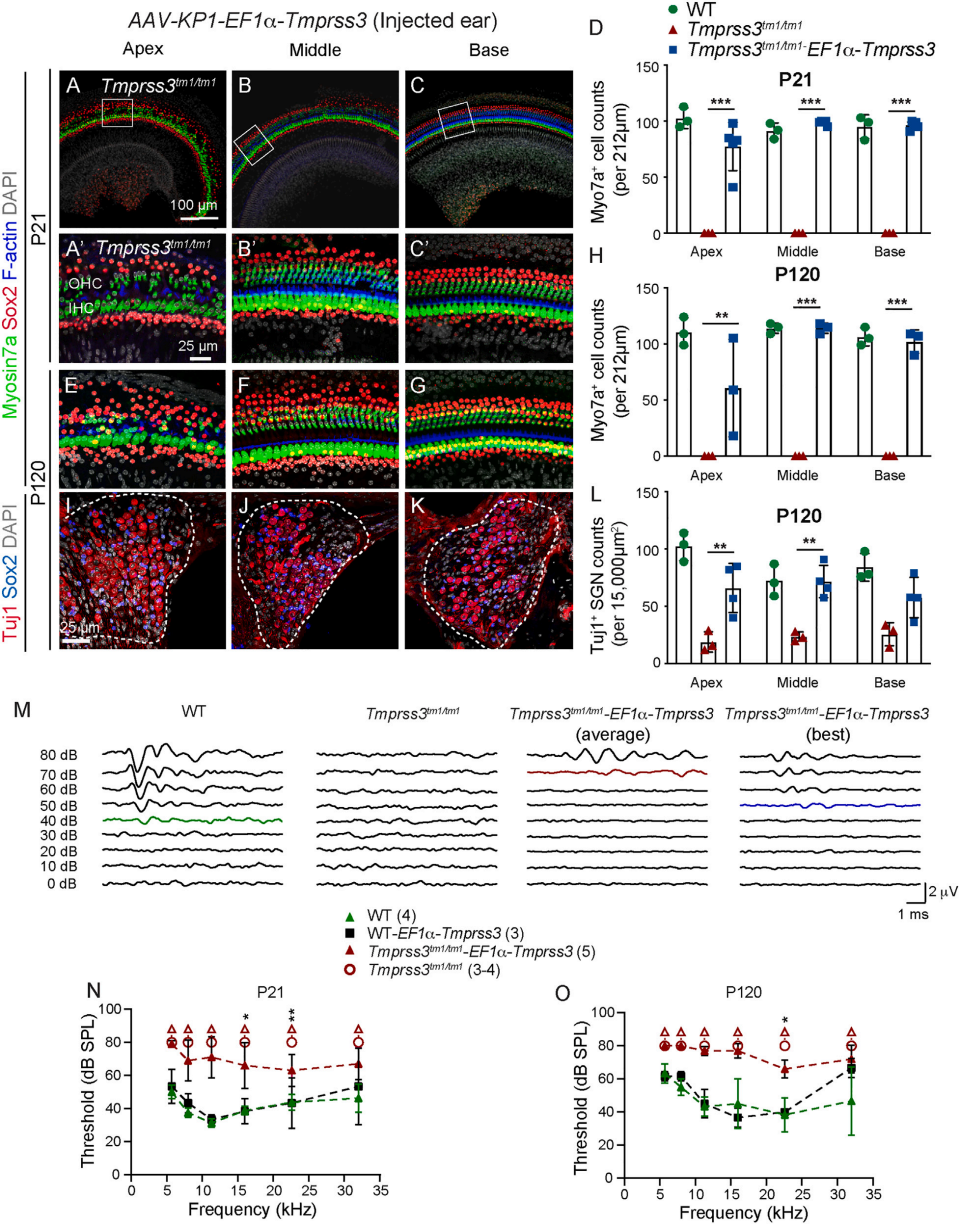
*AAV-KP1-EF1α-Tmprss3* partially prevents degeneration and auditory dysfunction in *Tmprss3*^*tm1/tm1*^ mice (A–C) After AAV-KP1-EF1α-*Tmprss3* injection (6.5 × 10^8^ vg) in P1 *Tmprss3*^*tm1/tm1*^ mice, most IHCs and OHCs were present in the middle and basal turns, while most IHCs and some OHCs remained in the apical turn at P21. (A′–C′) High-magnification images from (A)–(C). (D) Myo7a^+^ cell counts in each turn of treated *Tmprss3*^*tm1/tm1*^ cochlea were significantly higher than untreated *Tmprss3*^*tm1/tm1*^ cochlea and were similar to WT controls. (E–G) Hair cell survival persisted in each turn of P120-treated *Tmprss3*^*tm1/tm1*^ cochlea. (H) Each turn of P120-treated *Tmprss3*^*tm1/tm1*^ cochlea displayed significantly more hair cells than untreated *Tmprss3*^*tm1/tm1*^ cochlea and similar to WT controls. (I–K) Many SGNs were preserved in each turn of P120-treated *Tmprss3*^*tm1/tm1*^ cochlea, particularly the middle and basal turns. (L) Treated *Tmprss3*^*tm1/tm1*^ cochlea had higher SGN counts than untreated *Tmprss3*^*tm1/tm1*^ cochlea. (M) Raw ABR waveforms of P21 WT, untreated, and treated *Tmprss3*^*tm1/tm1*^ mice at 16kHz. (N and O). All treated *Tmprss3*^*tm1/tm1*^ ears demonstrated detectable ABR responses at P21 and P120, whereas untreated ears showed no responses. Data shown as mean ± SD. ∗p < 0.05, ∗∗p < 0.01, ∗∗∗p < 0.001, ∗∗∗∗p < 0.0001. Two-way ANOVA with Tukey’s multiple comparison. n = 3–5.

To assess its effects on hair cell degeneration and auditory dysfunction caused by *Tmprss3* deficiency, we administered *AAV-KP1-EF1α-Tmprss3* to P1 *Tmprss3*^*tm1/tm1*^ mice (Figure 4D). At P7, BaseScope *in situ* hybridization detected robust expression of *Tmprss3* transgene in all supporting cell subtypes and, to a lesser degree, inner and outer hair cells in the organ of Corti of *AAV-KP1-EF1α-Tmprss3*-injected *Tmprss3*^*tm1/tm1*^ cochlea (Figures S6A–S6D″). We observed preservation of both inner and outer hair cells in all three turns of the P21 *Tmprss3*^*tm1/tm1*^ cochlea (Figures 5A–5C). Nearly complete hair cell survival in the middle and basal turns was observed, whereas, in the apical turn, survival was partial and variable, especially of the outer hair cells. Myo7a^+^ cell counts in all three turns of treated *Tmprss3*^*tm1/tm1*^ cochlea were significantly higher than those in contralateral, control cochlea (p < 0.001; Figure 5D; Table S3). Surprisingly, three of five *Tmprss3*^*tm1/tm1*^ animals showed survival of some hair cells in the contralateral, control cochlea, suggesting a low level of transport of virus between ears (Figures S7A–S7D).

In *AAV-KP1-CAG-Tmprss3*-injected *Tmprss3*^*tm1/tm1*^ cochlea, we detected robust expression of *Tmprss3* transgene in both supporting cells and hair cells (Figures S6E–S6H″). Relative to *AAV-KP1-EF1α-Tmprss3*-injected *Tmprss3*^*tm1/tm1*^ cochlea, transgene appeared similarly expressed in supporting cells but higher among hair cells. How the level and pattern of transgene expression attributes to the lower cytotoxicity and ability of *AAV-KP1-EF1α-Tmprss3* to prevent hair cell degeneration is currently unclear.

We further examined whether hair cell survival in P120-treated *Tmprss3*^*tm1/tm1*^ cochleae persisted and whether degeneration of supporting cells and spiral ganglion neurons was also prevented. Similar to P21, sensory hair cells were consistently present in the middle and basal turns of the treated *Tmprss3*^*tm1/tm1*^ cochlea, with survival being more variable in the apical turn. Treated *Tmprss3*^*tm1/tm1*^ displayed significantly more hair cells than the contralateral, control cochlea (p < 0.01; Figures 5E–5H; Table S3). As seen at P21, one out of the five P120, *Tmprss3*^*tm1/tm1*^ mice had a partial rescue of hair cells in the contralateral ear (Figures S7E–S7H). Both the counts and organization of Sox2^+^ supporting cells in the treated *Tmprss3*^*tm1/tm1*^ cochlea were similar to those in the littermate and wild-type controls, while some disorganization was observed in the apex (Figures 5E–5G; Table S3). Furthermore, spiral ganglion neuron survival in the apical and middle turns was significantly higher in the treated *Tmprss3*^*tm1/tm1*^ cochlea than in the contralateral cochlea (p < 0.01, Figures 5I–L; Table S3).

Finally, all five *Tmprss3*^*tm1/tm1*^ mice injected with *AAV-KP1-EF1α-Tmprss3* showed detectable ABR responses across a range of frequencies at P21 and P120, when none of the non-injected *Tmprss3*^*tm1/tm1*^ mice or contralateral ears showed any responses at either age (Figures 5M–5O, S7M, S7N, and S7P; p < 0.0001). As expected, *AAV-KP1-EF1α-Tmprss3* did not cause ABR threshold shifts in wild-type animals (Figure 5N). As a group, injected ears of *Tmprss3*^*tm1/tm1*^ mice showed significantly higher thresholds than wild-type animals at P21 and P120 (Figures 5N, 5O, S7M, and S7P). Individually, each injected ear of *Tmprss3*^*tm1/tm1*^ mice had ABR responses detected (Figures S7N and S7Q). However, only one of the three injected *Tmprss3*^*tm1/tm1*^ ears showed detectable distortion product otoacoustic emission (DPOAE) responses, whereas none of the non-injected, mutant ears showed responses at P21 (Figure S7O). This suggests that outer hair cell function was not well restored even though most injected mutant ears showed improved survival of hair cells. In summary, these data indicate that treatment with *AAV-KP1-EF1α-Tmprss3* partially prevents hair cell degeneration and cochlear dysfunction.

## Discussion

*TMPRSS3* mutations cause DFNB8 and DFNB10 and are found in up to 11% of patients with sensorineural hearing loss.^4^^,^^36^ Previously, Guipponi and colleagues showed that *Tmprss3* null mice exhibit severe degeneration of cochlear hair cells between P12 and 14.^8^ Here, we found that *Tmprss3*^*tm1*^ mutant mice also demonstrated extensive inner and outer hair cell loss around P14,^32^ confirming that *Tmprss3* is required for hair cell survival after the onset of hearing. Strikingly, exogenous *Tmprss3* delivered via an rAAV vector using the KP1 capsid and the CAG promoter was toxic to multiple cell lines, caused hair cell loss in wild-type cochlea *in vivo*, and failed to prevent hair cell degeneration in *Tmprss3*^*tm1/tm1*^ mice. Toxicity was diminished when using lower viral titers or by replacing the KP1 capsid with DJ, neither of which prevented hair cell degeneration in *Tmprss3*^*tm1/tm1*^ mice. Finally, by using the EF1α promoter, exogenous *Tmprss3* was no longer cytotoxic *in vitro* and *in vivo*, prevented hair cell degeneration, and partially restored auditory function in *Tmprss3*^*tm1/tm1*^ mice. Recently, Du and colleagues reported similar findings of toxicity after overexpression of mouse *Tmprss3* in the cochlea.^37^

*Tmprss3* is broadly expressed in the embryonic and neonatal cochlea, with expression spanning sensory hair cells, a subset of spiral ganglion neurons, and non-sensory cells in the cochlea. This spatial pattern corroborates previous histological and single-cell RNA sequencing studies.^7^^,^^8^^,^^9^^,^^10^^,^^11^^,^^12^^,^^25^ Although TMPRSS3 is known to be a serine protease, its targets in the inner ear and exact function remain elusive.^15^ In this and previous studies, extensive loss of hair cells in *Tmprss3*-deficient mice indicates that *Tmprss3* is critical for their survival.

While we cannot rule out dysfunction of other *Tmprss3*-expressing cell types (e.g., supporting cells) in *Tmprss3*^*tm1/tm1*^ mice, their survival is likely less dependent on *Tmprss3*, as we did not detect significant supporting cell degeneration. Moreover, spiral ganglion neuron degeneration observed at P120 is likely attributed to a loss of trophic support from hair cell loss rather than *Tmprss3* deficiency.

In our study, high levels of exogenous *Tmprss3* induced cytotoxicity in multiple cell lines *in vitro* and in the juvenile cochlea *in vivo*. Outside the inner ear, *TMPRSS3* is highly expressed in several malignancies, including breast, ovarian, pancreatic, gastric, nasopharyngeal carcinoma, and brain gliomas.^38^^,^^39^^,^^40^^,^^41^^,^^42^^,^^43^ Several type II transmembrane serine proteases similar to TMPRSS3 are implicated in the development and progression of different types of cancer.^44^ Mechanistically, *Tmprss3* overexpression can decrease E-cadherin levels and thereby disrupt cell-cell adhesion, leading to tumor invasiveness and metastasis.^38^^,^^45^ Based on these studies, we postulate that *Tmprss3* overexpression similarly disrupts cell-cell adhesion, causing hair cells to be susceptible to degeneration after, but not prior to, the onset of hearing. Our model system has established a foundation to further investigate this potential mechanism.

Our study reveals that *AAV-KP1-EF1α-Tmprss3* was effective in partially preventing hair cell loss and improving auditory function in *Tmprss3*^*tm1/tm1*^ mice, although outer hair cell function was not well restored. *In situ* hybridization showed that expression of *Tmprss3* transgene was robust in supporting cells and remarkably lower in hair cells with the EF1α promoter. It is interesting that *Tmprss3* transgene expression in hair cells appeared higher after treatment with *AAV-KP1-CAG-Tmprss3* than *AAV-KP1-EF1α-Tmprss3* with expression in supporting cells comparable between the two promoters. While *AAV-KP1-EF1α-Tmprss3* partially rescued hearing function, *AAV-KP1-CAG-Tmprss3* did not and moreover was cytotoxic to cochlear cells. One interpretation is that the *Tmprss3* transgene is most critical for hair cell survival in the early postnatal cochlea, and the use of EF1α, and not CAG, promoter was able to restore *Tmprss3* expression early. Alternatively, it is possible that hair cell survival does not directly depend on expression of *Tmprss3* transgene within hair cells themselves but relies on an optimal level of expression in surrounding supporting cells, possibly via its effects on cell-cell junctions. Lastly, it is conceivable that a higher level of *Tmprss3* transgene expression is needed to restore outer hair cell function. By increasing the titer or using a promoter (other than CAG) that drives *Tmprss3* expression in outer hair cells, this may help to restore outer hair cell and overall cochlear function. Future work is necessary to delineate the function and downstream targets of Tmprss3 and the use of other promoters to drive *Tmprss3* transgene expression in the cochlea.

Most early studies evaluating the efficacy of inner ear gene therapy have focused on mutations affecting sensory hair cells,^17^^,^^46^^,^^47^ with some recent studies examining mutations affecting non-sensory cells within the cochlea.^48^^,^^49^^,^^50^ The current study suggests that overexpression of *Tmprss3* in supporting cells, rather than hair cells, may be more important for hair cell survival. However, this approach only partially prevents auditory function and fails to maintain outer hair cell function. While several studies have advocated the use of viral capsids with high transduction efficiency (e.g., AAV-ie, Anc80L65, and AAV2.7m8),^51^^,^^52^^,^^53^ it is unclear whether (1) broad transduction is necessary to rescue all cells expressing the gene of interest, (2) ectopic expression of a gene of interest can have deleterious effects in the inner ear, and (3) the level of transgene expression affects the efficacy of rescue. Even though AAV-KP1 robustly transduces multiple sensory and non-sensory cell types in the cochlea at rates comparable to several other viral capsids,^51^^,^^52^^,^^53^ *AAV-KP1-CAG-Tmprss3* caused death of cochlear hair cells. Both decreasing viral titers and packaging with the less efficient AAV-DJ capsid reduced cell death and cochlear dysfunction. While these results suggest that cytotoxicity depends on the high transduction efficiency of cochlear cells, both approaches failed to rescue the function and cellular morphology of *Tmprss3*^*tm1*^ mutant cochlea. By using an EF1α core promoter, we eliminated cytotoxicity in cell lines and in the cochlea *in vivo* and partially rescued hair cell degeneration and cochlear dysfunction. The findings that rescue of hair cell survival and auditory function was partial and not complete are likely multifactorial and may include differences in spatiotemporal expression between exogenous and endogenous *Tmprss3* as well as differences in the level of expression in cells of interest (e.g., hair cells). Nevertheless, since *AAV-EF1α-Tmprss3* led to sustained hair cell survival at least to P120, this approach is promising and should guide future studies to further optimize rescue of hearing loss caused by *Tmprss3* deficiency.

While some studies employed the CAG promoter within their viral vector constructs to successfully prevent cochlear dysfunction and degeneration in mouse models of hearing loss,^22^^,^^46^^,^^54^ this approach to drive *Tmprss3* expression leads to cytotoxicity, especially when used with the broadly transducing viral capsid KP1. In the retina, the input dose, viral capsid, the encoded gene, the promoter driving transgene expression, and target cells have been demonstrated to govern cellular toxicity related to AAV administration.^55^ Moreover, broadly active promoters were found to be more toxic to the retinal pigment epithelium, and a weaker photoreceptor-specific promoter attenuated the toxicity.^56^ Here, there are several possible contributing factors to cytotoxicity of *AAV-KP1-CAG-Tmprss3* in the cochlea, including ectopic transgene expression (e.g., stria vascularis, Reissner’s membrane), higher-than-native expression (hair cells and supporting cells), and differences in temporal expression. It is noteworthy that *CAG-Tmprss3* contained the woodchuck hepatitis virus post-transcriptional regulatory element (WPRE), while *EF1α-Tmprss3* did not. As WPRE has been reported to increase transgene expression,^57^ this sequence may further contribute to the toxicity observed. Lastly, *Tmprss3* appears more highly expressed in hair cells after treatment with *AAV-KP1-CAG-Tmprss3* than *AAV-KP1-EF1α-Tmprss3*, while that in supporting cells appears comparable between the two approaches. Thus, it is possible that the level of transgene expression in hair cells is more critical for hair cell function and in supporting cells for hair cell survival. The exact role of these factors warrants further investigation in future studies.

In summary, our results have demonstrated that the AAV-KP1 capsid has high transduction efficacy, but exogenous *Tmprss3* under the CAG promoter led to cytotoxic effects *in vitro* and *in vivo*. *Tmprss3*-induced cytotoxicity was ameliorated by reducing transduction efficacy across cochlear cells using the AAV-DJ capsid and by using the EF1α core promoter. Collectively, our data indicate that precise spatial and temporal control of *Tmprss3* expression is necessary for hair cell survival and cochlear function and further supports the need for a tailored approach to viral capsid and promoter selection to optimize gene therapy. These results may have important implications for the selection of viral capsids and promoters in human inner ear gene therapy.

## Materials and methods

### Mouse genotyping

The *Tmprss3*^*tm1/Lex*^ mouse (MMRRC lab, stock # 032680, background C57/Bl6) strain was used. The *Tmprss3*^*tm1/tm1*^ mouse was described as having an absence of a startle response to 120 dB (prepulse inhibition assay).^30^ Mice of both genders were used. Genomic DNA was prepared from mouse tail tips. The genomic DNA template was produced by adding 180 μL of 50 mM NaOH to tissue biopsies and incubating at 98°C for 1 h and then 15°C for 2 min. Next, 20 μL of 1 M Tris-HCl was added, and the samples were vortexed. The following primers were used: *Tmprss3* mutant forward (Fwd) (5′ GCA GCG CAT CGC CTT CTA TC), *Tmprss3* mutant reverse (Rev) (5′ CAG AGC CTT AAC TCT CCA CG), and *Tmprss3* wild-type Fwd (5′ TTC TAG GAC TTT GCT ATG ACC). All experiments were approved by the Institutional Animal Care and Use Committee (protocol #18606) at Stanford University.

### *In situ* hybridization

Previously published protocols were followed.^58^^,^^59^ Briefly, temporal bone tissues harvested from P1–P6 mice were fixed in 4% paraformaldehyde (in PBS, pH 7.4, Electron Microscopy Services) for 22 h at 4°C. The tissues were then cryoprotected using a serial sucrose gradient over 2 days starting at 15%, 20%, 30% sucrose solution and then gradually increasing the optimal cutting temperature compound (OCT) (Tissue Tek) gradient of 30% sucrose: 50% OCT, to 30:70, and finally to 100% OCT. Next, tissues were stored at −80°C until further use. The sections were cut at 10 μm thickness and placed on Superfrost Plus slides (Fisher).

Tissue sections were hybridized with commercial probes from Advanced Cell Diagnostics (ACDbio) and counterstained with hematoxylin (Sigma-Aldrich) according to the manufacturer’s instructions for fixed frozen sections with colorimetric detection. Briefly, sections were washed in PBS (1×) for 5 min and then treated with H_2_O_2_ for 10 min. Next, sections were permeabilized using target retrieval reagent (ACDbio) and proteinase before hybridization. RNAScope Red v2.5 kit (catalog #323350) was used with the following probes: *DapB* (catalog #310043), *Polr2a* (catalog #312471), and *Sox2* (catalog #401041). BaseScope v2 Red kit (catalog #323900) was used with the following probes: BaseScope Probe BA-Mm-Tmprss3-E1E2 (catalog #716911), BA-Mm-Ppib-1zz (catalog # 712351), BA-Dapb-1zz (catalog #701021) (ACDbio). The BaseScope Tmprss3 probe was diluted 1:20 and BaseScope step Amp 7 was performed for only 10 min to reduce signal intensity. *RNA Polymerase II* (*Polr2*) and *Peptidylprolyl Isomerase B* (*PPib*) are ubiquitously expressed in the cochlea and were selected as the positive controls. *DapB* gene is expressed by the *Bacillus subtilis* strain SMY, a soil bacterium, and not in mammalian tissues and was selected as a negative control (Figures S1C, S1D, S6G, and S6H). Wild-type and mutant cochleae were processed in parallel, with sections collected on the same slide and subjected to mRNA detection under identical conditions.

### Immunohistochemistry

Cochleae were harvested and fixed in 4% paraformaldehyde in PBS for 30–40 min for processing. Cochleae from P12–120 mice were decalcified with 120 mM EDTA for 24–120 h at 4°C. Whole mounts were dissected into three turns with the removal of Reissner’s and tectorial membranes, and the stria vascularis was carefully removed.

Cryosections were prepared as described above. Tissues were washed in 0.1% Triton X-100 (in PBS) × 3 for 5 min (cryosections) or 15 min (whole mounts) and then blocked with 5% donkey serum, 0.1% Triton-100, 1% bovine serum albumin, and 0.02% sodium azide (NaN_3_) in PBS at pH 7.4 for 1 h at room temperature. Primary antibody inoculation was then performed in the same blocking solution overnight at 4°C. The following primary antibodies were used: rabbit anti-Myosin7a (1:500–1:1,000; Proteus Bioscience), goat anti-Sox2 (1:200–1:400; Santa Cruz Biotechnology), and mouse anti-Tuj1 (1:1,000; Neuromics). The following day, tissues were re-permeabilized with 0.1% Triton X-100 in PBS and incubated with secondary antibodies diluted in PBS containing 0.1% Triton X-100, 1% bovine serum albumin, and 0.02% NaN_3_ for 2 h at room temperature. Fluorescent-conjugated phalloidin (1:1,000, Invitrogen), DAPI (1:10,000, Invitrogen), and Alexa Fluor secondary antibodies (488, 546 or 647, 1:250–1:500; Life Technologies) were then used. After washing with PBS for 3 × 10 min (cryosection) or 30 min (whole mount), tissues were mounted in either anti-fade fluorescent mounting medium (DAKO) or ProlongGold (Thermo Fisher, catalog #P10144) and coverslipped for imaging.

### Imaging and cell quantification

Whole mounts and cryosections were imaged as z stacks on Zeiss LSM700 and LSM880 (10× NA (numerical aperature) 0.3, 20× NA 0.8, and 40× NA 1.3 [oil]) confocal microscope. These images were captured at 1,024 × 1,024 (12-bit). Zen Black 2.3 (Carl Zeiss, Germany) was used.

For cell counting of whole-mount preparations, confocal images were analyzed using ImageJ software (NIH). Representative z stack images were taken on individual turns, and cells were counted from stacks and analyzed with ImageJ and Photoshop CS6 (Adobe Systems). For spiral ganglion neuron counting in sections, three sections per cochlea were used for quantification and averaged out (spiral ganglion neurons per 15,000 μm^2^).

### Assessment of hearing function

ABRs and DPOAEs were measured as previously described.^60^ Briefly, mice were anesthetized (100 mg/kg ketamine and 10 mg/kg xylazine) and injected intraperitoneally. Three needle electrodes were placed as follows: one inferior to the tympanic bulla, referenced to an electrode on the vertex of the head, with a ground electrode placed in the hindlimb. Tone pip stimuli were delivered at frequencies ranging from 4 to 46 kHz (4.0, 5.7, 8.0, 11.3, 16.0, 22.3, 32, 46.1 kHz) up to an 80-dB sound pressure level (SPL) in 10-dB steps. In all, 512 trials at each frequency and intensity were conducted and averaged. Distortion product otoacoustic emissions were measured by a probe tip microphone placed in the auditory canal. The sound stimuli used to elicit the DPOAE were two 1-s sine wave tones of differing frequencies (F2/F1 ratio = 1.22). Frequencies ranged from 5.7 to 32 kHz and the two tones were stepped up from 20 to 80 dB SPL in 10-dB increments. The amplitude of the cubic distortion product was 2xF1-F2. The threshold was calculated as a DPOAE of two standard deviations above the noise floor for each frequency. For analysis of ABR and DPOAE, thresholds were manually scored in a blinded fashion by two individuals (K.A.A. and P.J.A.). A lack of a response was designated at the highest sound level, 80 dB SPL.

### Vector constructs

The long isoform of *Tmprss3* cDNA is 2,874 bp and is therefore within the packing capacity (∼4.8 kbp) of rAAV vectors. The mouse *Tmprss3* gene was obtained from Origene (#MC216545) and cloned into the rAAV CAG-FLuc vector^32^ (plasmid #83281, Addgene) in place of the luciferase coding sequence using standard molecular biology techniques. The resulting construct (CAG-Tmprss3) contained AAV2 ITRs flanking the Tmprss3 sequence driven by a CAG promoter. A WPRE sequence between the coding sequence and the S40 late poly(A) signal was included to enhance expression.

Vector pAAV-EF1α-Tmprss3 was generated by replacing the respiratory syncytial virus (RSV) promoter region in plasmid pAAV-RHB^61^ with the first 212 nucleotides of the EF1α promoter from plasmid pAAV-EF1α-FLuc-WPRE-HGHpA (Addgene catalog #87951) using primers EF-core-NcoF (TATCTACCATGGGGCAGAGCGCACATCGCC) and EF-core-bluntR (CTGTGTTCTGGCGGCAAACCCG). Nco I and Ale I were used to ligate the promoter into the vector. The resulting vector, pAAV-EF1α-hAAT, was then digested with Ale I and Sal I to release the hAAT fragment. The *Tmprss3* gene was amplified from pAAV-CAG-Tmprss3 with primers Tmp-bluntF (TGGTGTGCACCTCCAAGCGCCACCATGGCCGCTTCAGA) and Tmp-SalR (TACTAGTCGACCCAGCTCAACCTCAAGTCTTCAGATCTCTCTC), digested with Sal I, and ligated into the vector backbone. For cloning of plasmid pAAV-EF1α-TdRed, the hAAT transgene in vector pAAV-EF1α-hAAT was replaced in a similar manner as described above with the TdRed sequence obtained by amplification from plasmid pAAV-CAG-tdTomato (Addgene catalog #59462) using TdRed-bluntF (ATCCGGTACCGCCACCATGGTG) and TdRed-SalR (AGCTGTCGACTTACTTATACAGCTCATCCATG). All plasmids were sequence verified using Sanger sequencing. The EF1α-Tmprss3 construct did not contain a WPRE sequence, and the SV40 poly(A) signal was replaced with the bovine growth hormone (bGH) poly(A) sequence. Expression was confirmed by western blotting with an antibody specific for mouse Tmprss3 (Proteintech, #1793-1-AP).

### rAAV production

293T/17 cells (ATCC #CRL-11268) were transfected with rep2-capKP1,^32^ the respective ITR-containing rAAV vector plasmid and pAd5 using the CaPO4 transfection method,^32^ or Transporter-5 transfection reagent (Polysciences, #26008). Virus was obtained from cell lysates 2 or 3 days post transfection and either purified using two rounds of CsCl centrifugation as previously described^32^ or using the AAV Pro All Serotype purification kit (Takara, #6666) according to the manufacturer’s instructions. Some rAAV preparations were further concentrated using Ultracel-100 spin columns (Millipore-Sigma, #UFC510008). Virus preparations were stored in aliquots at −80°C until use.

Viral genomes were isolated using the MinElute Virus Spin kit (Qiagen, #57704), and vector genome titers were determined using qPCR. For the Tmprss3-expressing rAAV preps, primers Tmp-qPCR-F (CACAGCAAGTACAAGCCAAAG) and Tmp-qPCR-R (GCTGGATGGT CTCGTCAAA) were used, while primer sets Td-qPCR-F (ATTACCTGGTGGAGTTCAAGAC) and Td-qPCR-R (GTCCTCGTTGTGTGAAGTGATA) were used to titer tdTomato-expressing rAAV preps. Copy number standards consisting of linearized and serially diluted (10^8^–10/μL) plasmids were included on each qPCR plate. All AAVs used in this study were made in the same facility (Kay lab), with batches with higher titers diluted down to match those with lower titers.

### *In vitro* transduction and transfection experiments

293T/17 cells or HeLa cells (ATCC #CCL-2) were seeded in 24-well plates, and, when they had reached a confluency of approximately 60%–70%, cells were transduced with rAAV at the MOI as indicated. Images were taken 3 days post transduction using a microscope with a built-in camera (Evos M5000, Invitrogen).

### Cell proliferation assays

293T/17 cells or HeLa cells were seeded in 96-well plates at a density of 10^4^ cells/well in 100 μL of medium and allowed to attach for 5 h. Cells were then transduced with rAAV diluted in 100 μL of medium in triplicate and assayed for proliferation at days 1, 2, 3, and 4 post transduction using the CellTiter 96 A_queous_ One Solution assay according to the manufacturer’s instructions (Promega, #G3581). Standard curves were obtained using nontransduced cells seeded at various densities. Cells that had been transduced with a huFIX-expressing rAAV packaged with the KP1 capsid as well as nontransduced cells were included as controls.

### *In vivo* gene transfer

To perform gene transfer experiments *in vivo*, P1 pups were anesthetized and injected via the posterior semicircular canal (PSCC) technique as previously described.^62^ Briefly, injection was performed using beveled glass microinjection pipettes, which were pulled from capillary glass on a P-2000 pipette puller (Sutter Instruments). Pups were anesthetized by rapid induction of hypothermia for 3–4 min on ice until loss of consciousness, and this state was maintained on a cooling platform for 10–15 min during the surgery. The surgical site was disinfected by scrubbing with Betadine and wiping with 70% ethanol. A postauricular incision was made to expose the PSCC and penetrate the tip of the micropipette. A total volume of 1 μL of either virus was unilaterally introduced at a rate of 300 nL/min into the left ear. The skin incision was closed using superglue. Body temperature was maintained on a 37°C warming pad for 30 min after surgery and before reintroduction into the parental cage.

### Statistical analyses

Data were analyzed using Microsoft Excel (Microsoft) and GraphPad Prism (GraphPad). Two-tailed Student’s t tests or analysis of variance with *post hoc* tests were used to calculate statistical significance. p < 0.05 was considered statistically significant. Data are shown as mean ± SD. For all experiments, n values represent the number of animals examined.

## Data Availability

Original data generated in this research are included in the main figures or supplementary items. Additional original data are available upon request.

## Supplemental information

Document S1. Figures S1–S9 and Tables S1–S3

Document S2. Article plus supplemental information
