# Supplementary material for: Selection of viral capsids and promoters affects the efficacy of rescue of Tmprss3-deficient cochlea
Source: Mol Ther Methods Clin Dev. 2023 Aug 11;30:413–28. doi: 10.1016/j.omtm.2023.08.004 (PMC10471831; doi:10.1016/j.omtm.2023.08.004)
Supplement: Document S1. Figures S1–S9 and Tables S1–S3 [file mmc1.pdf]

OMTM, Volume *30*

Supplemental information

Selection of viral capsids and promoters affects the efficacy of rescue of *Tmprss3*-deficient cochlea

Ksenia A. Aaron, Katja Pekrun, Patrick J. Atkinson, Sara E. Billings, Julia M. Abitbol, Ina A. Lee, Yasmin Eltawil, Yuan-Siao Chen, Wuxing Dong, Rick F. Nelson, Mark A. Kay, and Alan G. Cheng

Figure S1

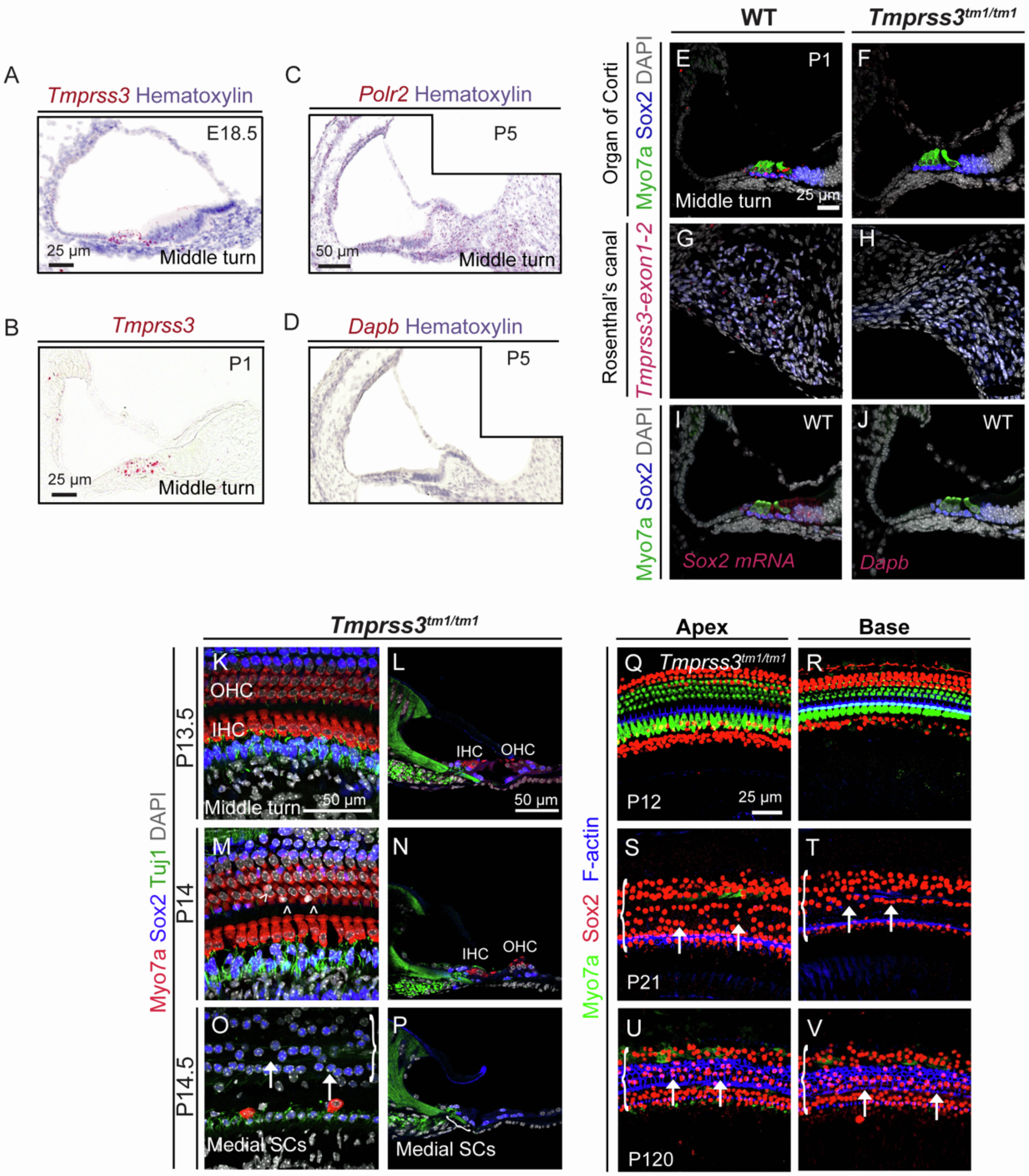

A
Tmprss3 Hematoxylin
E18.5
25 µm
Middle turn
B
Tmprss3
P1
25 µm
Middle turn
C
Polr2 Hematoxylin
P5
50 µm
Middle turn
D
Dapb Hematoxylin
P5
WT
Tmprss3tm1/tm1
Organ of Corti
Myo7a Sox2 DAPI
E
P1
Middle turn
25 µm
F
Rosenthal's canal
Tmprss3-exon1-2
G
H
Myo7a Sox2 DAPI
I
WT
Sox2 mRNA
J
WT
Dapb
Tmprss3tm1/tm1
Myo7a Sox2 Tuj1 DAPI
P13.5
K
OHC
IHC
Middle turn
50 µm
L
IHC
OHC
50 µm
P14
M
N
IHC
OHC
P14.5
O
Medial SCs
P
Medial SCs
Apex
Base
Myo7a Sox2 F-actin
Q
Tmprss3tm1/tm1
R
P12
25 µm
S
T
P21
U
V
P120

Figure S1. Characterization of *Tmprss3* expression and *Tmprss3*$^{tm1/tm1}$ cochlea. A-B. *In situ* hybridization (RNAScope) showing of *Tmprss3* mRNA expression in hair cell and supporting cell subtypes in the E18.5 and P1 wild-type cochlea (middle turn). **C-D.** Positive and negative controls for *in situ* hybridization using probes for *Polr2* and *Dapb* on the P5 wild-type cochlea (middle turn and counterstained with hematoxylin). **E-H**. *In situ* hybridization (BaseScope) detecting *Tmprss3* mRNA (exons 1-2) in wild-type, but not *Tmprss3* $^{tm1/tm1}$, organ of Corti and Rosenthal's canal (middle turn shown). **I-J.** *Sox2* mRNA and not *Dapb* was detected in the wild-type cochleae. **K-P.** Whole mount and section of *Tmprss3*$^{tm1/tm1}$ cochleae showing pyknotic nuclei (arrowheads) at P14 prior to loss of IHCs and OHCs at P14.5 with disorganization of supporting cells (marked with parenthesis, middle turn shown). **Q-V.** Whole-mount *Tmprss3*$^{tm1/tm1}$ mice showing loss of IHCs and OHCs and disorganized supporting cells (marked with parentheses) after P12 (apical and basal turns shown). IHC, inner hair cell; OHC, outer hair cell; WT, wild-type.

Figure S2

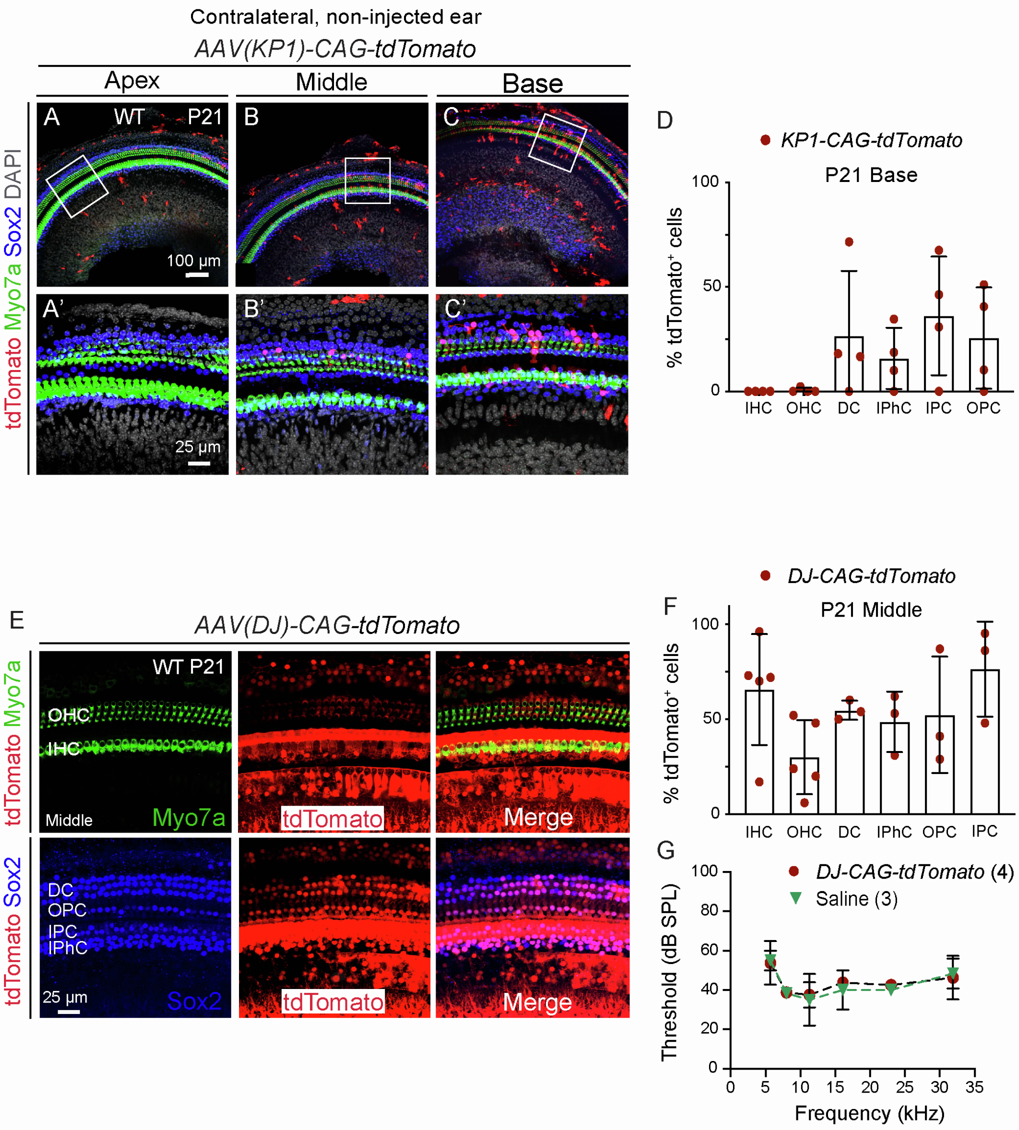
Contralateral, non-injected ear
AAV(KP1)-CAG-tdTomato
Apex
Middle
Base
A
WT
P21
B
C
100 µm
A'
B'
C'
25 µm
tdTomato Myo7a Sox2 DAPI
D
KP1-CAG-tdTomato
P21 Base
% tdTomato+ cells
IHC
OHC
DC
IPhC
IPC
OPC
E
AAV(DJ)-CAG-tdTomato
WT P21
OHC
IHC
Middle
Myo7a
tdTomato
Merge
tdTomato Myo7a
DC
OPC
IPC
IPhC
25 µm
Sox2
tdTomato Sox2
F
DJ-CAG-tdTomato
P21 Middle
G
DJ-CAG-tdTomato (4)
Saline (3)
Threshold (dB SPL)
Frequency (kHz)

Figure S2. Transduction with *AAV-KP1-CAG-tdTomato* and *AAV-DJ-CAG-tdTomato*. A-C. Representative images of P21 contralateral, non-injected wild-type cochleae, showing few labeled supporting cells, especially in the basal region. **A'-C'** are high magnification images from **A-C**. **D.** Quantification of $tdTomato^+$ cells in the P21 basal turn. **E.** P21 cochleae that were injected with *AAV-DJ-CAG-tdTomato* at P1 demonstrated many $tdTomato^+$ sensory and supporting cells. **F.** Quantification of $tdTomato^+$ sensory and supporting cells in the middle turn of P21 cochleae. **G.** ABR showing comparable ABR thresholds among *AAV-DJ-CAG-tdTomato*- and saline-injected P21 animals. Data shown as mean±S.D. n = 3-5. IHC, inner hair cell; OHC, outer hair cell; DC, Deiters' cell; IPhC, inner phalangeal cell; IPC, inner pillar cell; OPC, outer pillar cell; WT, wild-type.

Figure S3

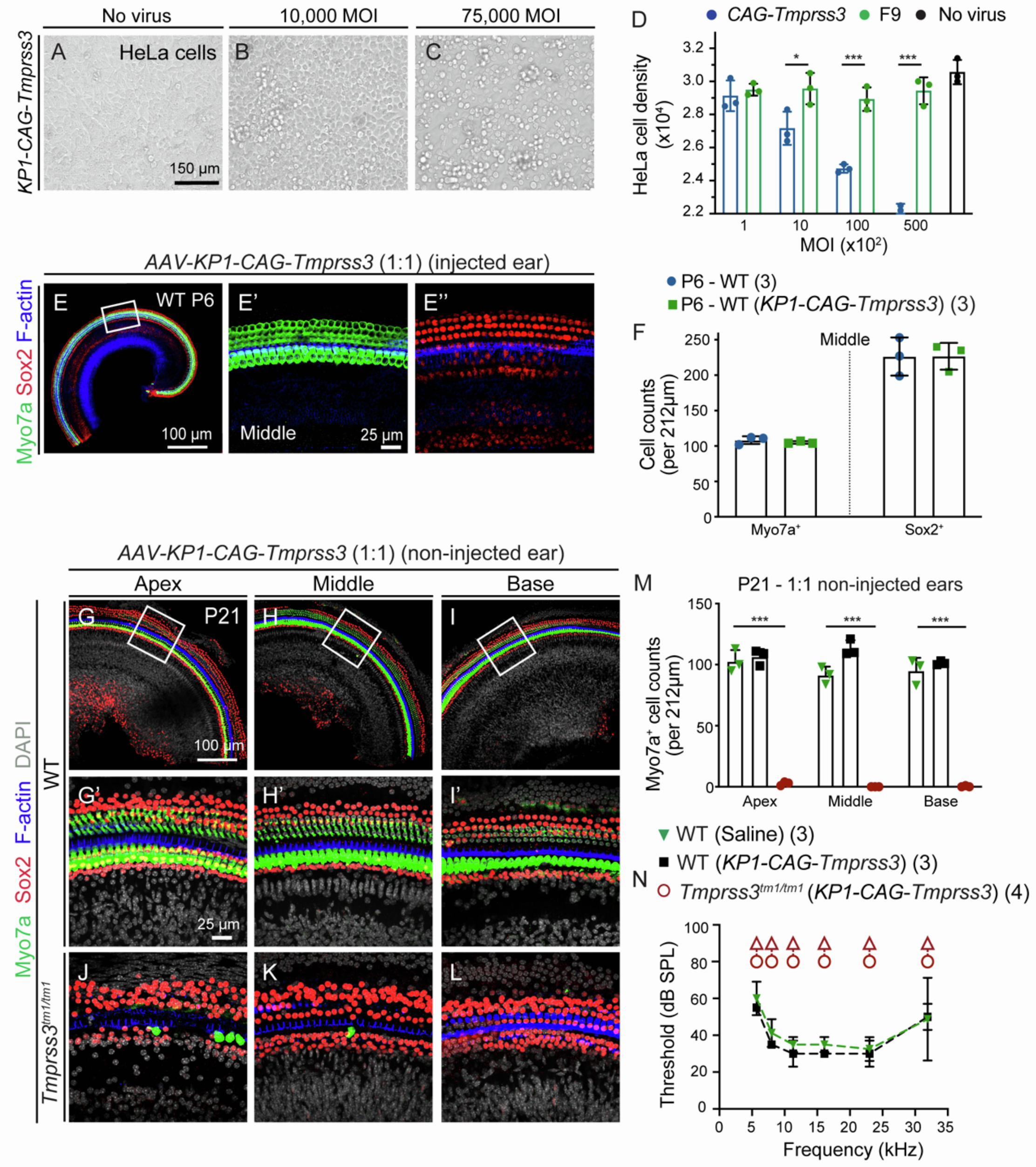

No virus
10,000 MOI
75,000 MOI
KP1-CAG-Tmprss3
A
HeLa cells
B
C
150 µm
D
CAG-Tmprss3
F9
No virus
HeLa cell density (x10⁴)
MOI (x10²)
AAV-KP1-CAG-Tmprss3 (1:1) (injected ear)
Myo7a Sox2 F-actin
E
WT P6
E'
E''
100 µm
Middle
25 µm
P6 - WT (3)
P6 - WT (KP1-CAG-Tmprss3) (3)
F
Cell counts (per 212µm)
Myo7a⁺
Sox2⁺
AAV-KP1-CAG-Tmprss3 (1:1) (non-injected ear)
Apex
Middle
Base
Myo7a Sox2 F-actin DAPI
WT
Tmprss3tm1/tm1
G
H
I
P21
G'
H'
I'
J
K
L
M
P21 - 1:1 non-injected ears
Myo7a⁺ cell counts (per 212µm)
WT (Saline) (3)
WT (KP1-CAG-Tmprss3) (3)
N
Tmprss3tm1/tm1 (KP1-CAG-Tmprss3) (4)
Threshold (dB SPL)
Frequency (kHz)

Figure S3. Cytotoxicity of *AAV-KP1-CAG-Tmprss3*. A-C. Transduction of HeLa cells using *AAV-KP1-CAG-Tmprss3* caused cell death and detachment, especially at a high MOI (multiplicity of infection). **D.** Proliferation assay demonstrated that increasing the MOI of *AAV-KP1-CAG-Tmprss3* significantly decreased HeLa cell viability. Controls used no virus or huF9 expressing rAAV. **E-E''.** Injection of *AAV-KP1-CAG-Tmprss3* (2.0×10^8 vg) at P1 caused no obvious cell loss in the P6 wild-type cochlea. **F.** Quantification of $Myo7a^+$ hair cells and $Sox2^+$ supporting cells in non-injected and injected P6 wild-type cochleae. **G-I.** Contralateral, non-injected ears of P21 wild-type mice injected with *AAV-KP1-CAG-Tmprss3* (1:1 titer, 2.0×10^8 vg) at P1, showing no evidence of hair cell or supporting cell degeneration. **G'-I'** are high magnification images from **G-I. J-L.** Severe hair cell degeneration in the contralateral, non-injected ears of P21 $Tmprss3^{tm1/tm1}$ mice. **M.** Quantification of $Myo7a^+$ hair cells in the contralateral, non-injected ears of wild-type (saline or virus-injected) and $Tmprss3^{tm1/tm1}$ mice. **N.** ABR thresholds were similar between contralateral, non-injected ears of P21 wild-type mice injected with saline or *AAV-KP1-CAG-Tmprss3*, whereas those of $Tmprss3^{tm1/tm1}$ mice showed no responses. Data shown as mean±S.D. *$p<0.05$, ***$p<0.001$. Two-way ANOVA with Tukey's multiple comparison. n = 3-4. WT, wild-type.

Figure S4

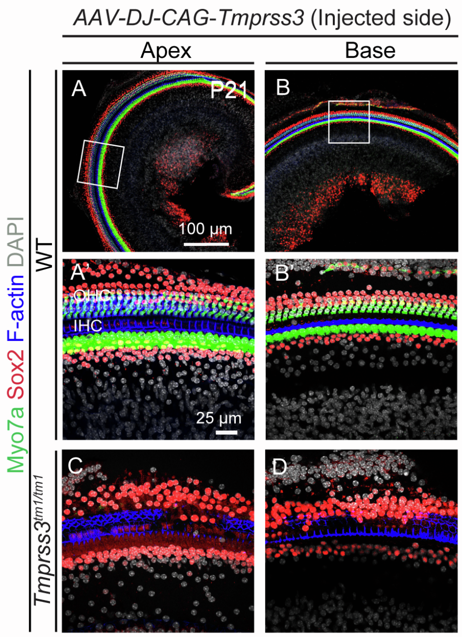

AAV-DJ-CAG-Tmprss3 (Injected side)
Apex
Base
A
P21
B
100 µm
A'
OHC
IHC
25 µm
B'
C
D
Myo7a Sox2 F-actin DAPI
WT
Tmprss3^tm1/tm1

Figure S4. Effects of *AAV-DJ-CAG-Tmprss3 in vivo*. A-B. No cell loss was detected in the P21 wild-type cochlea after *AAV-DJ-CAG-Tmprss3* had been injected at P1 (2.0x10^8 vg) (apex and base shown). **A'-B'** represent high magnification images from **A-B**. **C-D.** Sensory hair cell loss was not prevented by *AAV-DJ-CAG-Tmprss3* in *Tmprss3*$^{tm1/tm1}$ mice.

Figure S5

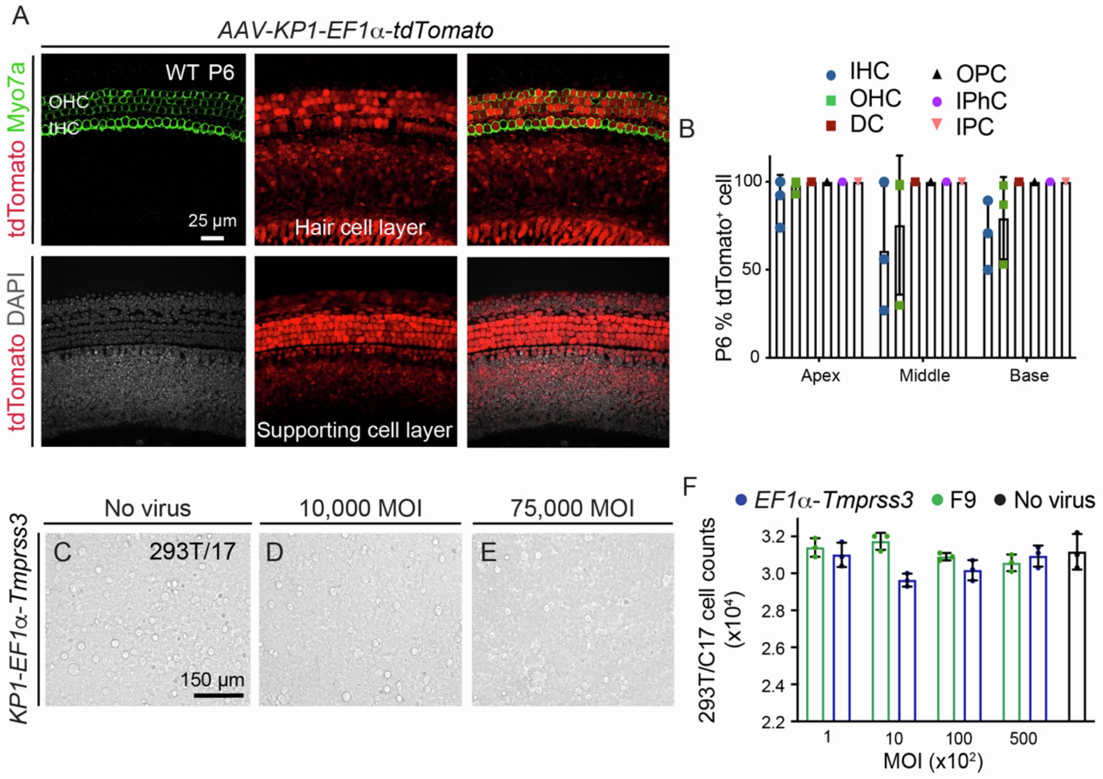

A
AAV-KP1-EF1α-tdTomato
WT P6
OHC
IHC
tdTomato Myo7a
25 µm
Hair cell layer
tdTomato DAPI
Supporting cell layer
B
IHC
OHC
DC
OPC
IPhC
IPC
P6 % tdTomato+ cell
100
50
0
Apex
Middle
Base
No virus
10,000 MOI
75,000 MOI
KP1-EF1α-Tmprss3
C
293T/17
D
E
150 µm
F
EF1α-Tmprss3
F9
No virus
293T/C17 cell counts (x10⁴)
3.2
3.0
2.8
2.6
2.4
2.2
1
10
100
500
MOI (x10²)

Figure S5. *AAV-KP1-EF1α-Tmprss3 does not cause cytotoxicity*. A. After injection with *AAV-KP1-EF1α-tdTomato* ($1.0x10^9$ vg) at P1, there was robust tdTomato expression in both sensory and supporting cells in the P6 cochlea (middle turn shown). **B.** Quantification showing transduction of most IHCs and OHCs and almost all supporting cell subtypes in each cochlear turn at P6. **C-E.** Transduction of HEK cells using *AAV-KP1-EF1α-Tmprss3* did not result in cell death even at 60 hours after transduction and at different MOIs compared to the control (no virus). **F.** Increasing the MOI of *AAV-KP1-EF1α-Tmprss3* did not decrease the viability of 293T/17 cells. Controls used no virus or huF9 expressing rAAV. Data shown as mean±S.D. Two-way ANOVA with Tukey's multiple comparison. n = 3-4. IHC, inner hair cell; OHC, outer hair cell; DC, Deiters' cell; IPhC, inner phalangeal cell; IPC, inner pillar cell; OPC, outer pillar cell; SGN, spiral ganglion neuron; WT, wild-type.

Figure S6

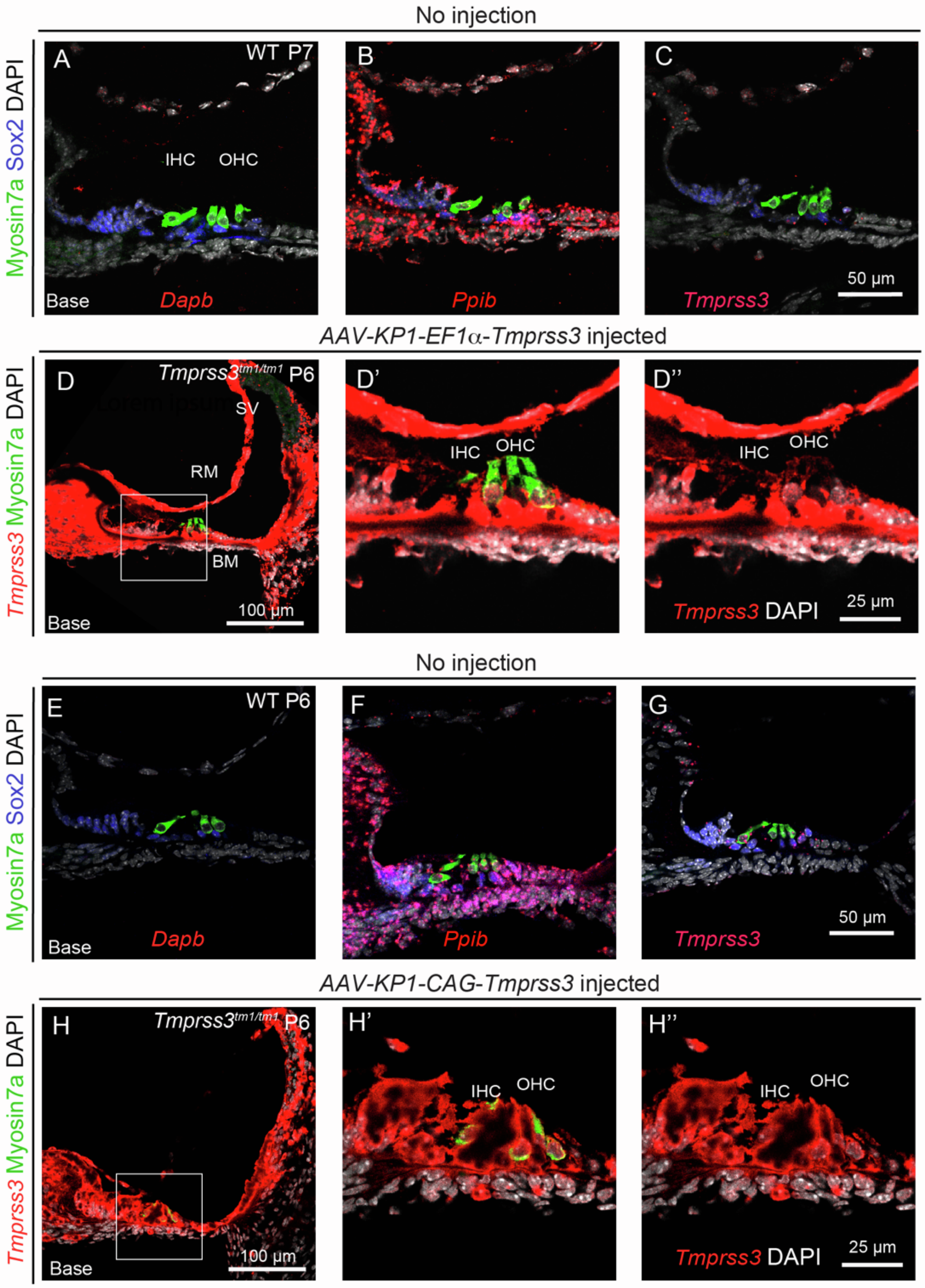
No injection
Myosin7a Sox2 DAPI
A
WT P7
IHC
OHC
Base
Dapb
B
Ppib
C
Tmprss3
50 μm
AAV-KP1-EF1α-Tmprss3 injected
Tmprss3 Myosin7a DAPI
D
Tmprss3^tm1/tm1 P6
SV
RM
BM
Base
100 μm
D'
IHC
OHC
D''
IHC
OHC
Tmprss3 DAPI
25 μm
No injection
Myosin7a Sox2 DAPI
E
WT P6
Base
Dapb
F
Ppib
G
Tmprss3
50 μm
AAV-KP1-CAG-Tmprss3 injected
Tmprss3 Myosin7a DAPI
H
Tmprss3^tm1/tm1 P6
Base
100 μm
H'
IHC
OHC
H''
IHC
OHC
Tmprss3 DAPI
25 μm

Figure S6. Spatial expression of Tmprss3 transgene in treated *Tmprss3*$^{tm1/tm1}$ mutant cochlea. A-B. Positive and negative controls for BaseScope *in situ* hybridization performed on cochlear sections from P7 wild-type mice. **C**. *Tmprss3* transcripts were detected in the organ of Corti. **D**. After injection of *AAV-KP1-EF1*α*-Tmprss3* at P1, robust expression of *Tmprss3* was detected in the organ of Corti, spiral ligament, spiral limbus, greater epithelial ridge, Reissner's membrane (RM), and lateral cochlear wall in the P7 *Tmprss3*$^{tm1/tm1}$ cochlea. **D-D"**. High magnification image showing high expression of *Tmprss3* transgene in supporting cells, low expression in hair cells. **E-F**. Positive and negative controls for BaseScope *in situ* hybridization performed on cochlear sections from P6 wild-type mice. **G**. *Tmprss3* transcripts were detected in the organ of Corti. **H**. After injection of *AAV-KP1-CAG-Tmprss3* at P1, robust expression of *Tmprss3* was detected in the organ of Corti, spiral ligament, spiral limbus, greater epithelial ridge, Reissner's membrane (RM), and lateral cochlear wall including the stria vascularis in the P6 *Tmprss3*$^{tm1/tm1}$ cochlea. **H-H"**. High magnification image showing high expression of *Tmprss3* transgene in hair cells and supporting cells. Shown are representative images from 2-3 animals.

Figure S7

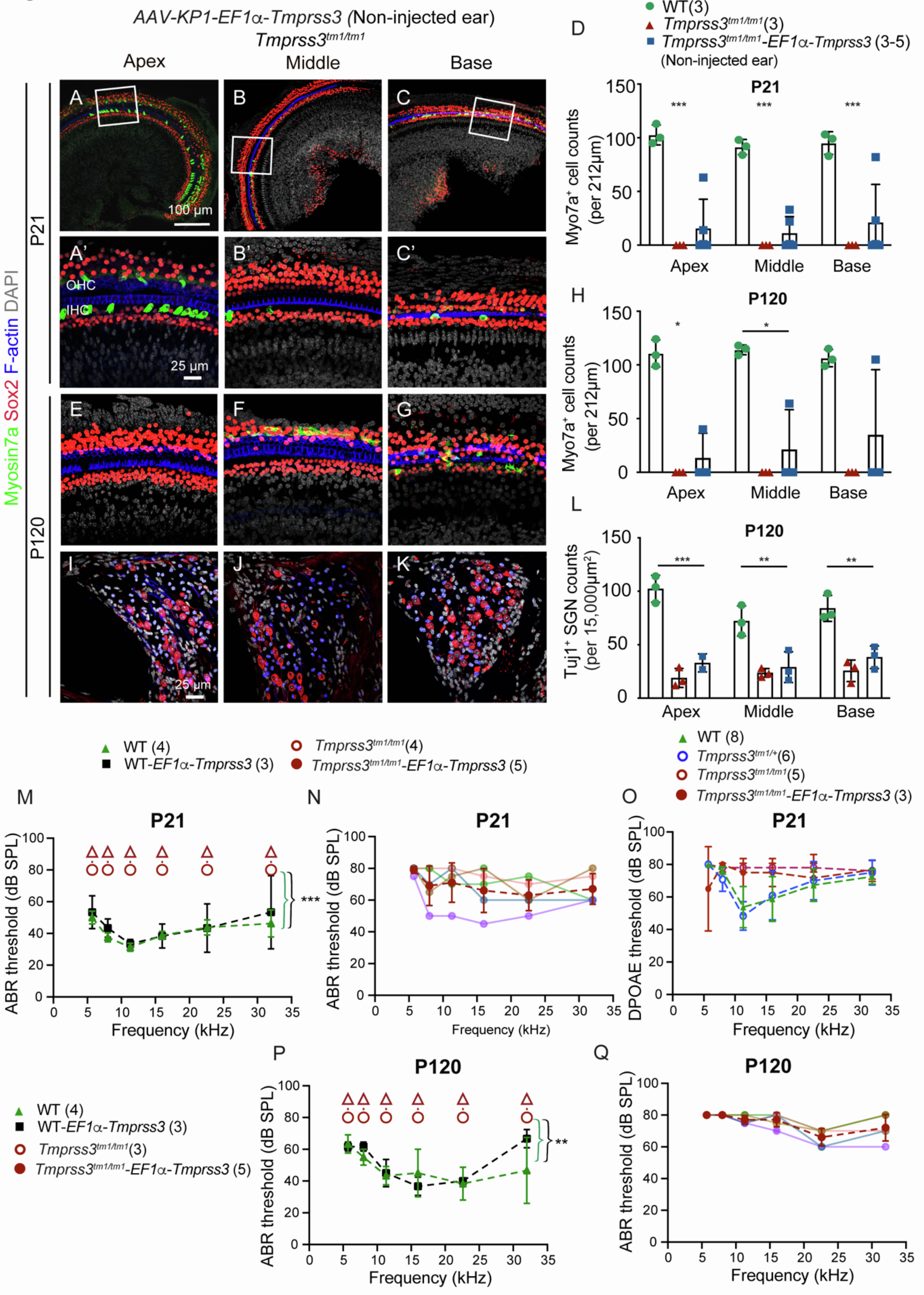

AAV-KP1-EF1α-Tmprss3 (Non-injected ear)
Tmprss3tm1/tm1
Apex
Middle
Base
P21
P120
Myosin7a Sox2 F-actin DAPI
100 µm
25 µm
OHC
IHC
WT(3)
Tmprss3tm1/tm1(3)
Tmprss3tm1/tm1-EF1α-Tmprss3 (3-5)
(Non-injected ear)
Myo7a+ cell counts (per 212µm)
Tuj1+ SGN counts (per 15,000µm2)
WT (4)
WT-EF1α-Tmprss3 (3)
Tmprss3tm1/tm1(4)
Tmprss3tm1/tm1-EF1α-Tmprss3 (5)
WT (8)
Tmprss3tm1/+(6)
Tmprss3tm1/tm1(5)
Tmprss3tm1/tm1-EF1α-Tmprss3 (3)
ABR threshold (dB SPL)
DPOAE threshold (dB SPL)
Frequency (kHz)
Tmprss3tm1/tm1(3)

Figure S7. ***AAV-KP1-EF1α-Tmprss3* partially prevents degeneration and auditory dysfunction in *Tmprss3*$^{tm1/tm1}$ mice. A-C.** Few hair cells were present in the non-injected cochlea of P21 *Tmprss3*$^{tm1/tm1}$ mice that received *AAV-KP1-EF1α-Tmprss3* in the contralateral ear at P1 (apical and basal turns shown). **A'-C'** are high magnification images from **A-C**. **D.** Myo7a^{+} hair cell counts of non-injected ears of wild-type and *Tmprss3*$^{tm1/tm1}$ mice. Some non-injected cochleae of *Tmprss3*$^{tm1/tm1}$ animals, which were injected in the contralateral ears, showed hair cell survival. **E-G.** Rare hair cells were observed in the non-injected cochlea of P120 *Tmprss3*$^{tm1/tm1}$ mice. **H.** Quantification of Myo7a^{+} hair cells in non-injected ears of wild-type and *Tmprss3*$^{tm1/tm1}$ mice. **I-L.** SGN degeneration in the non-injected ears of the treated P120 *Tmprss3*$^{tm1/tm1}$ mice was similar to that in the untreated *Tmprss3*$^{tm1/tm1}$ cochlea. **M.** ABR thresholds of P21 wild-type and *Tmprss3*$^{tm1/tm1}$ mice. **N**. ABR thresholds of individual P21 *Tmprss3*$^{tm1/tm1}$ mice administered *AAV-KP1-EF1α-Tmprss3* at P1. **O.** DPOAE thresholds of P21 wild-type and *Tmprss3*$^{tm1/tm1}$ mice. **P**. ABR thresholds of P120 wild-type and *Tmprss3*$^{tm1/tm1}$ mice. **Q**. ABR thresholds of individual P120 *Tmprss3*$^{tm1/tm1}$ mice administered *AAV-KP1-EF1α-Tmprss3* at P1. Data shown as mean±S.D. *$p<0.05$, **$p<0.01$, ***$p<0.001$. Two-way ANOVA with Tukey's multiple comparison. n = 3-5. IHC, inner hair cell; OHC, outer hair cell; WT, wild-type; SGN, spiral ganglion neuron.

Supplemental Figure 8

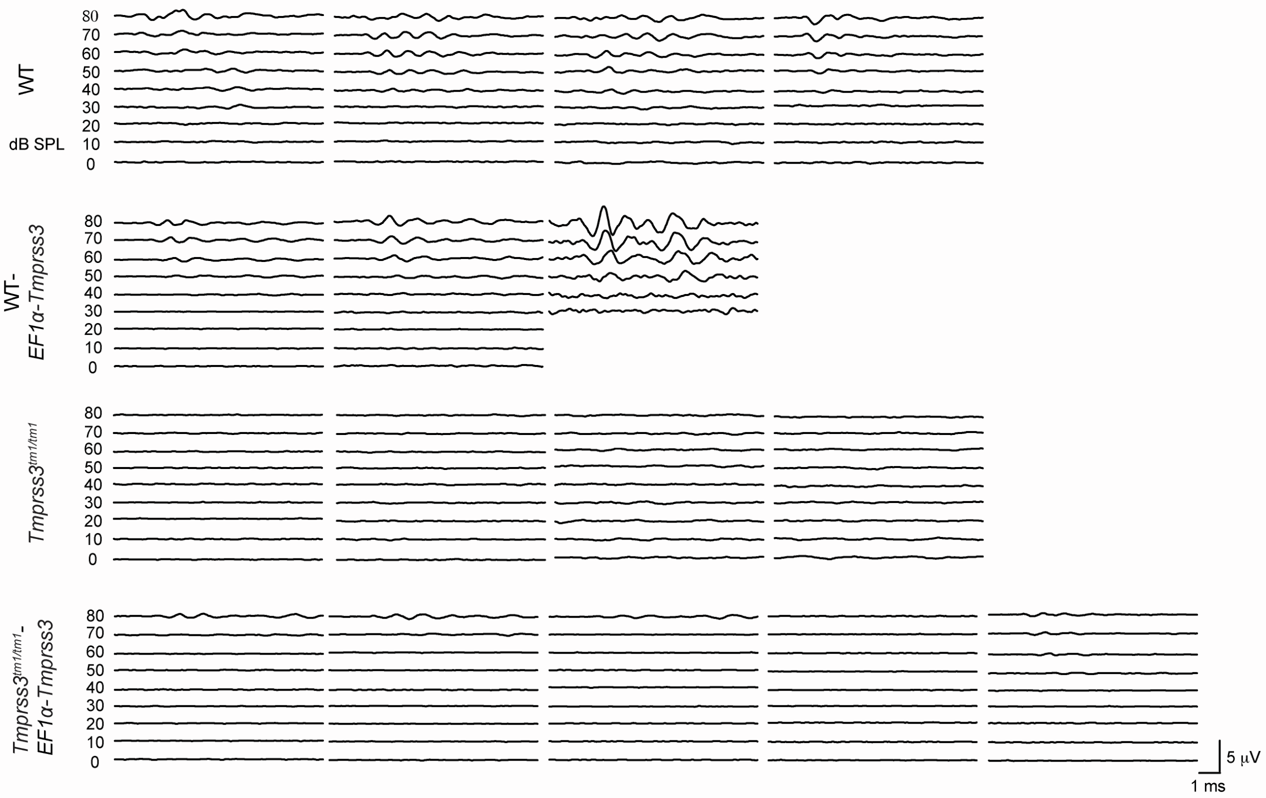

Figure S8. ABR tracings from P21 wildtype and *Tmprss3*$^{tm1/tm1}$ mice that were untreated or treated with *AAV-KP1-EF1*α*-Tmprss3*.

Supplemental Figure 9

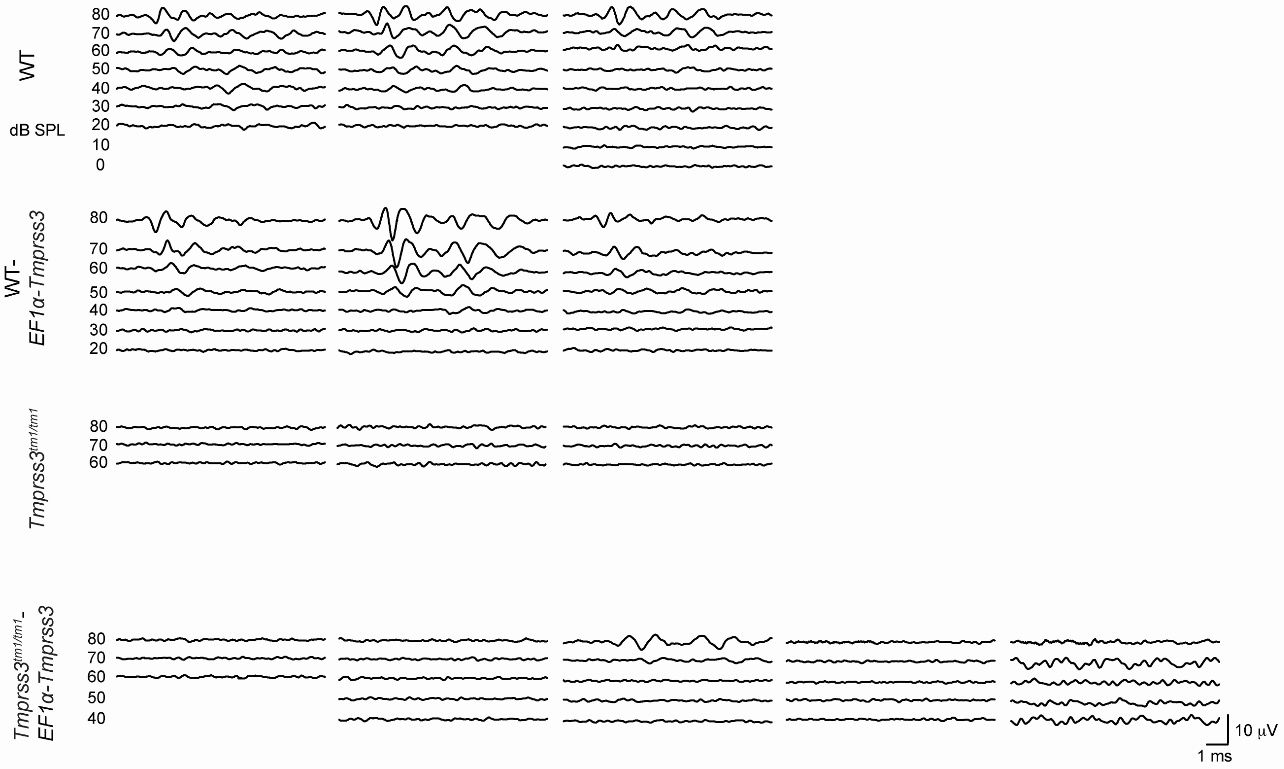

Figure S9. ABR tracings from P120 wildtype and *Tmprss3*$^{tm1/tm1}$ mice that were untreated or treated with *AAV-KP1-EF1*α*-Tmprss3*.

Table S1. Quantification of hair cells, supporting cells, and spiral ganglion neurons

Age	Cell Type	WT			*Tmprss3*$^{tm1/+}$			*Tmprss3*$^{tm1/tm1}$		
		Apex	Mid	Base	Apex	Mid	Base	Apex	Mid	Base
**P5 (3)**	IHC	25.2±1.3	24.7±0.8	n/a	26.1±1.5	25.6±0.8	n/a	23.9±1.3	26.5±2.7	n/a
	OHC	96.3±4.0	94.5±2.0	n/a	92.8±2.7	97.2±2.8	n/a	99.8±6.7	96.7±13.9	n/a
	DC/OPC	130.3±5.4	122.8±9.9	n/a	122.3±9.4	121.5±3.3	n/a	121.9±6.9	113.6±5.4	n/a
	IPC/IPhC	107.8±2.0	113.1±0.8	n/a	105.2±2.0	104.2±4.6	n/a	105.6±7.3	98.9±4.7	n/a
**P12 (3-4)**	IHC	25.3±1.9	27.5±2.4	27.5±1.7	25.6±0.6	26.0±2.0	28.3±1.6	26.0±0.0	28.3±0.6	26.0±1.0
	OHC	83.8±1.5	85.3±5.4	87.5±10.6	91.0±1.7	79.3±1.5	90.0±2.0	89.3±4.0	81.3±4.7	85.0±9.2
	DC/OPC	147.3±10.4	133.7±15.8	115.3±19.5	133.0±7.0	125.7±5.1	145.0±10.0	140.7±6.8	120.7±11.1	135.0±15.1
	IPC/IPhC	146.3±70.2	132.0±41.8	140.3±42.8	133.3±59.9	113.7±28.7	121.3±10.5	173.6±18.9	131.3±3.2	129.0±9.5
**P21 (3-4)**	IHC	22.0±2.6	22.3±3.5	21.6±2.5	24.0±1.7	24.3±1.5	24.3±2.1	0±0.0***, ###	0±0.0***, ###	0±0.0***, ###
	OHC	69.7±5.7	69.7±10.7	73.3±12.9	79.7±6.1	80.0±4.6	78.3±4.0	0±0.0***, ###	0±0.0***, ###	0±0.0***, ###
	DC/OPC	126.3±8.5	121.0±10.1	108.0±9.8	131.7±13.6	118.0±22.3	108.3±3.2	104.0±24.8	108.8±17.9	105.8±20.7
	IPC/IPhC	106.3±32.6	104.0±19.1	82.3±25.9	124.7±19.4	119.0±18.5	120.3±18.9	106.2±11.5	112.4±16.0	101.5±22.4
	SGN	92.0±2.6	80.0±15.9	111.3±10.0	93.3±23.9	85.7±11.2	110.3±8.4	83.4±0.8	81.0±11.5	106.7±27.8
	Glia	114.3±16.9	90.7±11.2	103.3±12.5	113.3±21.4	97.7±13.3	113.7±20.3	110.3±20.5	97.0±9.2	127.7±29.0
**P120 (3-6)**	IHC	26.7±1.5	27.7±3.1	25.3±2.1	25.7±0.6	27.7±2.1	27.0±1.7	2.3±2.5***, ###	0±0.0***, ###	0±0.0***, ###
	OHC	88.7±7.2	86.3±5.0	82.3±6.5	79.3±6.1	83.0±2.0	84.3±10.5	1.7±1.5***, ###	0±0.0***, ###	0±0.0***, ###
	DC/OPC	82.0±16.1	83.3±20.5	118.3±8.5	95.7±37.1	86.0±26.0	110.5±33.2	39.3±38.9*	64.6±45.2	109.7±18.2
	IPC/IPhC	94.8±31.0	97.5±15.1	102.0±2.6	107.3±21.1	110.3±21.4	106.0±10.4	31.0±26.9**, #	58.2±37.4*	88.0±15.0
	SGN	110.7±8.3	92.7±5.5	77.9±8.4	106.7±6.7	99.0±11.5	78.0±19.0	30.8±24.6*, ##	24.0±4.0*, #	29.8±11.7***,###
	Glia	77.0±10.5	80.8±10.9	57.7±4.7	65.7±8.9	89.0±15.6	79.0±11.5	62.9±20.7	58.4±14.3	51.9±15.3***

Shown are counts from whole mount cochlea (IHC, OHC, DC/OPC-lateral, IPC/IPhC-medial per 212 μm) and cryosection (SGN, Glia per 15,000 μm^2) from P5, 12, 21, and 120 wild-type, *Tmprss3*$^{tm1/+}$, *Tmprss3*$^{tm1/tm1}$ mice. Mean ± SD. Number of animals listed in parentheses.

*Represents significant difference across time points within the same genotype.

#Represents significant difference among age-matched *Tmprss3*$^{tm1/tm1}$ and wild-type or *Tmprss3*$^{tm1/+}$ mice.

*p<0.05, **p<0.01, ***p<0.001 (two-way ANOVA followed by post-hoc analysis via Tukey's multiple comparisons test).

##p<0.01, ###p<0.001 (two-way ANOVA followed by post-hoc analysis via Tukey's multiple comparisons test).

n/a = not available

IHC, inner hair cell; OHC, outer hair cell; DC, Deiters' cell; IPhC, inner phalangeal cell; IPC, inner pillar cell; OPC, outer pillar cell; SGN, spiral ganglion neuron; WT, wild-type.

Table S2. Viral transduction efficiency of cochlear cell types.

Age	Cell Type	WT injected			WT contralateral		
		Apex	Mid	Base	Apex	Mid	Base
AAV-KP1-CAG-tdTomato (1.0 x 10^9 vg)							
**P6 (3)**	IHC	0.0±0.0	0.0±0.0	0.0±0.0			
	OHC	0.0±0.0	6.6±11.5	0.0±0.0			
	DC	100.0±0.0	85.0±25.9	84.7±23.2			
	OPC	100.0±0.0	100.0±0.0	100.0±0.0			
	IPC	100.0±0.0	100.0±0.0	100.0±0.0			
	IPhC	100.0±0.0	100.0±0.0	100.0±0.0			
**P21 (5)**	IHC	100.0±0.0	92.9±10.3	90.4±8.3	0.0±0.0	0.0±0.0	0.0±0.0
	OHC	100.0±0.0	88.2±14.9	84.0±14.0	0.0±0.0	0.0±0.0	0.0±0.0
	DC	100.0±0.0	100.0±0.0	100.0±0.0	0.0±0.0	6.3±10.9	26.6±31.2
	OPC	100.0±0.0	100.0±0.0	100.0±0.0	0.0±0.0	2.3±4.0	25.5±24.3
	IPC	100.0±0.0	100.0±0.0	100.0±0.0	0.0±0.0	15.0±25.9	36.2±28.4
	IPhC	100.0±0.0	99.6±0.9	100.0±0.0	0.0±0.0	27.0±46.8	15.8±14.6
	SGN	68.7±11.7	61.7±16.0	34.0±7.5			
AAV-DJ-CAG-tdTomato (1.0 x 10^9 vg)							
**P21 (4)**	IHC	57.0±33.8	65.6±29.3	33.0±18.0			
	OHC	17.7±17.0	30.0±19.5	9.6±4.1			
	DC	46.7±6.8	54.6±5.0	42.3±6.1			
	OPC	50.7±29.9	52.3±30.2	29.3±6.0			
	IPC	87.7±4.1	76.4±25.0	80.6±42.2			
	IPhC	85.3±20.4	48.7±15.9	21.3±9.3			
AAV-KP1-EF1α-tdTomato (1.0 x 10^9 vg)							
**P6 (3)**	IHC	91.6±12.3	61.0±36.8	70.0±19.6			
	OHC	97.9±3.4	75.4±39.5	79.3±23.4			
	DC	100.0±0.0	100.0±0.0	100.0±0.0			
	OPC	100.0±0.0	100.0±0.0	100.0±0.0			
	IPC	100.0±0.0	100.0±0.0	100.0±0.0			
	IPhC	100.0±0.0	100.0±0.0	100.0±0.0			
**P21 (3)**	IHC	0.0±0.0	19.3±33.5	0.0±0.0	0.0±0.0	0.0±0.0	0.0±0.0
	OHC	100.0±0.0	98.5±2.6	95.8±4.5	0.0±0.0	0.0±0.0	0.0±0.0
	DC	100.0±0.0	85.0±25.9	84.7±23.2	0.0±0.0	0.0±0.0	10.3±17.9
	OPC	100.0±0.0	100.0±0.0	100.0±0.0	0.0±0.0	0.0±0.0	10.7±17.6
	IPC	100.0±0.0	100.0±0.0	100.0±0.0	0.0±0.0	0.0±0.0	37.8±41.6
	IPhC	100.0±0.0	100.0±0.0	100.0±0.0	0.0±0.0	0.0±0.0	18.1±16.8

Shown are percent tdTomato labeled cells from whole mount cochlea (IHC, OHC, DC, OPC, IPC, IPhC per 212 µm) and cryosection (SGN per 15,000 µm^2) from P6 and 21 wild-type mice. Mean ± SD. Number of animals listed in parentheses.

Table S3. Survival of hair cells, supporting cells, and spiral ganglion neurons

Age	Cell Type	WT			*Tmprss3*$^{tm1/tm1}$		
		Apex	Mid	Base	Apex	Mid	Base
		AAV-KP1-CAG-Tmprss3 **(1.98x10^8 vg)**					
**P6 (3)**	Myo7a$^+$	107.7±4.0	108.3±5.5	n/a			
	Sox2$^+$	231.7±7.6	226.3±27.0	n/a			
**P21 (3) Saline**	Myo7a$^+$	102.7±9.3	91.3±7.0	95.0±10.6			
	Sox2$^+$	220.6±38.9	230.7±22.3	201.3±44.1			
**P21 1:1 (3)**	Myo7a$^+$	53.3±2.1***	51.0±19.1**	58.3±21.1*	4.0±1.0$^{\#\#\#}$	1.0±1.0$^{\#\#\#}$	0.0±0.0$^{\#\#\#}$
	Sox2$^+$	217.0±19.3	213.0±16.1	189.7±56.1	201.7±31.0	222.0±6.6	206.7±42.1
**P21 1:2 (3)**	Myo7a$^+$	110.3±8.3	105.3±9.6	111.7±4.7	2.0±2.0$^{\#\#\#}$	1.0±1.0$^{\#\#\#}$	1.3±2.3$^{\#\#\#}$
	Sox2$^+$	224.3±20.6	187.3±3.1	211.0±17.1	179.7±16.2	172.7±29.6	165.0±8.7
**P21 1:10 (3)**	Myo7a$^+$	104.7±5.5	96.3±2.1	93.7±8.5	2.7±0.6$^{\#\#\#}$	0.7±0.6$^{\#\#\#}$	0.0±0.0$^{\#\#\#}$
	Sox2$^+$	224.3±35.6	230.0±26.2	212.3±20.5	220.7±24.8	223.0±24.6	202.0±33.8
		AAV-DJ-CAG-Tmprss3 **(1.92x10^8 vg)**					
**P21 (3)**	Myo7a$^+$	111.0±3.6	105.7±4.0	108.3±6.0	0.0±0.0$^{\#\#\#}$	1.3±2.3$^{\#\#\#}$	0.0±0.0$^{\#\#\#}$
	Sox2$^+$	175.3±4.7	176.3±2.5	173.0±7.9	164.0±34.4	162.3±7.5	156.0±6.8
		AAV-KP1-EF1α-Tmprss3 **(6.5x10^8 vg)**					
**P21 (3-5)**	Myo7a$^+$	104.0±7.9	97.3±4.5	101.0±9.6	77.4±21.5	98.8±2.2	96.6±3.6
	Sox2$^+$	201.3±17.8	210.0±45.3	193.7±8.6	186.6±12.6	178.2±20.3	178.0±15.5
		**Saline**			*AAV-KP1-EF1α-Tmprss3* **(6.5x10^8 vg)**		
**P120 (3-5)**	Myo7a$^+$	110.7±12.5	114.0±4.6	106.3±8.1	60.7±43.5	96.7±18.8	102.0±10.4
	Sox2$^+$	192.7±21.4	187.3±41.0	204.3±21.1	229.7±60.5	224.0±13.0	223.3±31.1
	TuJ1$^+$	102±12.5	72.2±14.1	84.0±12.2	66.0±21.4	71.7±14.2	57.7±17.6

Shown are counts from whole mount cochlea (Myo7a$^+$: IHC, OHC; Sox2$^+$: DC, OPC, IPC, IPhC per 212 μm) and cryosection (Tuj1$^+$: SGN per 15,000 μm^2) from P6, 21, and 120 wildtype, *Tmprss3*$^{tm1/+}$, *Tmprss3*$^{tm1/tm1}$ mice. Mean ± SD. Number of animals listed in parentheses.
*Represents significant difference with saline-injected controls.
$^{\#}$Represents significant difference among age-matched *Tmprss3*$^{tm1/tm1}$ and wildtype mice injected with the same viral vector.
*p<0.05, **p<0.01, ***p<0.001 (two-way ANOVA followed by post-hoc analysis via Tukey's multiple comparisons test).
$^{\#\#}$p<0.01, $^{\#\#\#}$p<0.001 (two-way ANOVA followed by post-hoc analysis via Tukey's multiple comparisons test).
